# Gaboxadol increases resting theta and alpha power without affecting evoked responses in fragile X syndrome in a home-based setting

**DOI:** 10.1186/s11689-026-09700-5

**Published:** 2026-05-08

**Authors:** Lisa A. De Stefano, Hyeonseok Kim, Craig A. Erickson, Lauren M. Schmitt, Kelli C. Dominick, Ernest V. Pedapati, Richard Huckle, Robert Wilson, Walker S. McKinney, Debra L. Reisinger, Meredith N. Nelson, Ashley E. Dapore, Paul S. Horn, Makoto Miyakoshi

**Affiliations:** 1 Division of Child and Adolescent Psychiatry, Cincinnati Children’s Hospital Medical Center, Cincinnati, OH USA; 2 Department of Psychiatry and Behavioral Neuroscience, University of Cincinnati College of Medicine, Cincinnati, OH USA; 3 Phelan-McDermid Syndrome Foundation, Osprey, FL 34229 USA; 4 HealX Ltd., Cambridge, UK; 5 Division of Developmental and Behavioral Health, Children’s Mercy Hospital, Kansas City, MO USA; 6 Department of Pediatrics, University of Missouri Kansas City School of Medicine, Kansas City, MO USA; 7 Division of Behavioral Medicine and Clinical Psychology, Cincinnati Children’s Hospital Medical Center, Cincinnati, OH USA; 8 Department of Pediatrics, University of Cincinnati College of Medicine, Cincinnati, OH USA; 9 Division of Neurology, Cincinnati Children’s Hospital Medical Center, Cincinnati, OH USA

## Abstract

**Background:**

Fragile X syndrome (FXS) lacks FDA-approved treatments despite various small molecules contributing to phenotypic rescue in the *FMR1* knockout (KO) mouse model. Translation from the mouse model has been hampered by phenotypic heterogeneity that contributes to participation barriers among participants who are most affected and may be unable to regularly visit the research laboratory. The current study utilized a crossover design to test the acute neural and behavioral effects of a single 10 mg dose of gaboxadol and the reliability of electroencephalography (EEG) and behavioral data collected in participant homes compared to the clinic.

**Methods:**

Ten adult males with full mutation FXS completed four blinded dosing visits (two placebo, two gaboxadol), with two occurring in-home and two in-lab. Pre- and post-dose assessments included resting high-density EEG, an auditory chirp paradigm, RBANS List Learning, and NIH Toolbox Cognition Battery subtests.

**Results:**

No serious adverse events were reported. Compared with placebo, gaboxadol increased theta and alpha band power, with no interaction between collection environment (home vs. lab). Additionally, gaboxadol increased the proportion of electrodes with detectable low-frequency peaks and slowed the peak frequency. There were no effects on auditory-evoked measures or NIH Toolbox, with only a marginal effect on RBANS List Learning. An analysis of pre-dose EEG found reliability estimates across testing locations for all tested resting power and behavioral measures that were similar to in-lab reliability estimates found in the literature.

**Conclusions:**

Single-dose gaboxadol augmented theta and alpha power in FXS during resting EEG, similar to previous findings in the typically developing population and in the *FMR1* KO, without normalizing gamma abnormalities, altering auditory-evoked responses, or contributing to behavioral change. These results did not significantly differ between the home and lab settings, supporting the feasibility of in-home data collection for clinical trials in FXS, including those that use complex measures such as EEG as endpoints.

**Trial registration:**

clinicaltrials.gov, NCT06334419, Registration Date: March 8, 2024.

**Supplementary Information:**

The online version contains supplementary material available at 10.1186/s11689-026-09700-5.

## Introduction

Fragile X syndrome (FXS) is the most common inherited form of intellectual disability and autism spectrum disorder, with a clinical phenotype that is additionally often marked by anxiety, attentional symptoms, sensory hypersensitivity and irritability, among other concerns. FXS is caused by a CGG triplet repeat expansion in the promoter region of the FMR1 gene resulting in gene methylation and subsequent silencing of gene transcription with resultant reduction or absence of fragile x messenger ribonucleoprotein (FMRP) expression. Despite phenotypic rescue with various small molecules in the *Fmr1* knockout (KO) mouse model of FXS, there are no FDA-approved treatments for FXS, and translation from the mouse model has been largely unsuccessful. One potential reason for this lack of translation is the increase in phenotypic heterogeneity in participants with FXS, who do not have uniform repeat counts and methylation patterns like the *Fmr1* KO mouse, a deletion animal model. This variability highlights the need to use biomarkers that are sensitive across a broad range of clinical severity, particularly in individuals with low FMRP expression who may more closely resemble the *Fmr1* KO model.

Many of our previous studies of drug response in patients with FXS have utilized a double-blind, randomized, single-dose crossover design, with participants visiting the clinic to engage in pre- and post-dose encephalography (EEG) in response to placebo and study drug. This approach allows us to assess target engagement utilizing cross-species validated biomarkers such as increased gamma power at rest [14, 16, 26, 44, 60] and altered intertrial phase coherence (ITC) during auditory stimulation [10, 11, 16, 26, 34, 40]. Recently published trials have found that baclofen reduced gamma power across-species [17], while lovastatin and minocycline did not normalize EEG in humans [30]. This design holds promise for early, rapid screening of potential drugs that engage neural targets relevant to FXS prior to more costly, intensive chronic dosing trials.

However, the requirement that participants travel to often distant clinic sites for repeated visits to engage in clinical trial research can provide a barrier to participation. This barrier may be particularly challenging to overcome among participants who present with significant impairment who may be best modeled by work with the *Fmr1* KO mouse and could potentially benefit the most from clinical trial translational medicine efforts. For example, in our recent study of baclofen, follow-up analyses found the strongest reductions in gamma power in participants who had the lowest FMRP [17], and thus were likely to be more severely affected [4, 25]. Home-based trial protocols may help overcome this barrier, improving access to clinical research and representation of more severely affected individuals.

The current study aimed to expand upon our single-dose design by collecting pre- and post-dose EEG and behavioral measures both in home and in clinic in response to a candidate study drug, gaboxadol following a 10 mg single dose. Gaboxadol was primarily developed for the treatment of insomnia. Its clinical development involved over 4300 subjects across Phase I, II, and III studies, demonstrating efficacy in improving sleep maintenance and initiation, increasing slow-wave sleep (SWS), and showing a generally favorable safety and tolerability profile with no significant next-day residual or withdrawal effects at therapeutic doses (up to 15 mg for adults and 10 mg for the elderly administered as a single dose before sleep to insomnia patients). However, the development for the insomnia indication was terminated in March 2007 due to its effect size for sleep onset not being considered competitive and an unattractive risk–benefit ratio at higher doses. The dosing of 10 mg per occasion in this current study was therefore deemed appropriate and safe.

Gaboxadol is an orthosteric agonist at GABA_A_ receptors, with high selectivity for extrasynaptic receptors containing δ subunits [57], which contribute to tonic inhibition in multiple neuronal populations [52]. Previous examinations of the effects of gaboxadol on EEG power spectra have chiefly occurred during non-REM sleep, with most studies reporting an increase in slow wave (or delta, 1–4.5 Hz) and theta activity (4–5–7 Hz) in a dose-dependent manner [27–29, 59]. Mixed results have been found in the alpha band, with one study reporting a decrease in alpha power [28] while another found increased alpha power at the highest studied dose (20 mg [27]). Interestingly, multiple studies that examined sex found that these effects were strongest in females, potentially due to altered δ subunit density [8, 28], although several studies conducted in both young and elderly subjects suggested a modest but significant increase (approximately 10 to 20%) in weight adjusted plasma exposure between female and male subjects.

Previous studies of gaboxadol in the *Fmr1* KO mouse found it to rescue inhibitory tone and increase action potential thresholds to that of wild-type (WT) mice in amygdalar slices [42]. Behaviorally, gaboxadol has been shown to reduce aberrant pre-pulse inhibition at low decibels in vivo [41] and normalize a variety of behaviors, particularly at a low dose [6]. However, a recent attempt to find electrophysiological correlates in *Fmr1* KO mice did not find a normalizing impact of single-dose gaboxadol on gamma power or visually- or auditory-evoked potentials, though gaboxadol at its higher dose was associated with increased power broadly from 1–30 Hz [15]. In humans, a clinical trial of gaboxadol in 23 individuals with FXS evaluated three fixed doses in parallel groups across a 12-week dosing period and found that 60% of participants showed clinical improvement [5], though no neural biomarkers were assessed and there was no control group. As a result, it remains unclear whether gaboxadol engages relevant neural targets in humans with FXS.

In the current study, we sought to determine not only the effect of single-dose gaboxadol on EEG biomarkers commonly found in FXS, but also the feasibility and consistency of collecting these biomarkers in the participants’ home environment. By extending the single-dose crossover design into participant homes, we aimed to assess whether decentralized EEG collection could support early-phase biomarker-driven drug screening in FXS, which has the potential to reduce participant burden, improve participation rates, and better represent the FXS phenotype in clinical research.

## Methods and materials

### Study design

This study consisted of two in-home and two in-clinic dosing visits with a two-week washout period between visits. Participants always began the sequence with a clinic visit at which inclusion and exclusion criteria were assessed prior to the first dose, followed by a home visit, another in-clinic visit, and a final home visit. All participants received two doses of placebo (one of each at each location) and two doses of 10 mg gaboxadol (one of each at each location) with all investigators and participants blinded to treatment status. The study protocol was conducted in accordance with the Declaration of Helsinki, approved by the Cincinnati Children’s Hospital Medical Center Institutional Review Board, and registered at clinicaltrials.gov with the identifier NCT06334419.

Potential participants were deemed eligible if they: (1) were male, aged 18–40; (2) had confirmed full *Fmr1* mutation (> 200 CGG repeats); (3) were in generally good health at the time of the initial clinic visit, as determined by a study physician; (4) if receiving serotonin-selective reuptake inhibitor (SSRIs), serotonin-norepinephrine reuptake inhibitor (SNRI), or serotonin antagonist and reuptake inhibitor (SARI), were on a stable, well-tolerated dose for the previous three months with no further changes anticipated; (5) were not sexually active or confirmed at least one form of contraceptive. Study exclusion criteria are described in Supplement S1. Additionally, participants were required to be open to completing research visits at a non-clinic location, either in-home or at a location of their choice. Initial recruitment efforts focused on participants within 100 miles from the medical center, but this criteria was relaxed to allow timely recruitment, resulting in a visit range of approximately 300 miles.

At the baseline visit, which served as the first dosing day, participants completed demographic, phenotypic, and health history forms, received a physical examination and Clinical Global Impressions – Severity score from a study physician, and completed the Abbreviated Stanford Binet 5 [49]. At each clinic visit, participants received a blood draw and urinalysis to assess health status and optionally donated a post-dose pharmacokinetic blood sample to assess the amount of gaboxadol in the blood. Laboratory work results were reviewed prior to dosing at first visit. At every dosing visit, including both clinic and home visits, participants completed the following measures pre- and post-dose: EEG, Repeatable Battery for the Assessment of Neuropsychological Status (RBANS) List Learning [47], and portions of the NIH Toolbox Cognition Battery [61],Oral Reading Recognition, Picture Vocabulary, Speeded Matching, Pattern Comparison Processing Speed, Flanker Inhibitory Control and Attention, and Dimensional Change Card Sort subtests). Post-dose measures were collected 60–90 min after administration of the drug in alignment with the Tmax of gaboxadol [43].

### Procedures

#### Electroencephalography

EEG recordings were obtained using saline-based 128-channel HydroCel Geodesic Sensor Nets connected to an EGI Net Amps 400 amplifier (Magstim EGI, Eugene, OR). Data were sampled at 1000 Hz at collection. All EEG assessments were collected pre- and post-dose at each visit. For home visits, the EEG amplifier was transported in the secure Pelican case in which it is initially shipped by the manufacturer, and set up by trained clinical research coordinators following a validated standard operating procedure. Given the initial home visit was always the second visit of the study, research coordinators were aware of the participant’s EEG net size and packed both a primary and backup net, with preference for reusing the same net as was used for laboratory data collection.

#### Rest

Ten minutes of resting-state EEG data were collected while participants sat in a quiet room and watched a silent movie of their choosing on an iPad to facilitate participation.

#### Chirp

Participants additionally listened to the “chirp” stimulus, a 2-s white noise carrier stimulus that is amplitude modulated by a sinusoid that linearly increases in frequency from 0–100 Hz over its duration. Chirp stimuli were presented 200 times at 65 db SPL through headphones with an intertrial interval that randomly varied between 1.5–2.5 s.

#### EEG preprocessing

EEGLAB was used for EEG preprocessing [7]. After data import, resting data were downsampled to 250 Hz while chirp data were not downsampled. Following this step, a high-pass filter was applied with a 0.5 Hz cutoff frequency, a 1.0 Hz transition band, and a Blackman window. Line noise at 60 Hz and its harmonics were removed using the CleanLine plugin [3, 35, 38]. Then, the first and last 3 s of each recording were trimmed. To detect and reject artifactual channels, the continuous data were first segmented into 1,000 equal-length epochs. To ensure equal epoch length, a small number of trailing samples were discarded from the end of each recording. For each channel, the maximum absolute amplitude was computed across time within each epoch. To model channel-wise amplitude distributions, a generalized extreme value (GEV) distribution was fit to the maximum absolute amplitude across epochs. A per-channel threshold was then defined as the 90th percentile of the fitted GEV distribution. Epochs exceeding this threshold were marked as high-amplitude outliers. Standard deviations were then computed across time within each epoch, and epochs with high-amplitude outliers were excluded from further estimation. Channels whose median standard deviation across non-outlier epochs fell below 0.1 µV were classified as flat and excluded from the montage. Subsequently, the remaining data were re-referenced to the median (computed after excluding outlier time points). Standard deviation was again calculated per epoch per channel, and a robust threshold was applied to the median SD values to identify channels with excessively large amplitude fluctuations. Specifically, channels whose median standard deviation exceeded the group-level median by more than 20 times the scaled median absolute deviation (MAD × 1.4826) were rejected. A similar approach was used in our previous study [34]. Artifact Subspace Reconstruction (ASR) was applied to remove transient high-amplitude artifacts [22, 33],T. R. [39]. ASR was run with a burst criterion of 25, using Euclidean distance. Calibration was restricted to clean data segments by setting BurstCriterionRefMaxBadChns to 0, ensuring that no channels exceeded the artifact threshold during calibration [19, 20]. Previously rejected channels were restored by spherical spline interpolation. The original reference channel (Cz), which is not recorded separately in the EGI system, was restored as a zero-filled channel to preserve its position in the montage. All channels, including the reconstructed Cz, were then re-referenced to the average reference [21].

#### NIH toolbox cognition battery (NIH Toolbox)

Participants completed six subtests from the NIH Toolbox that have previously been assessed for use in FXS [53]. For each measure, the pre-dose change sensitive score was subtracted from the post-dose change sensitive score to form a difference score that was then assessed statistically. The number of participants included in each assessment varies across visit date, as some participants were nonverbal (*n* = 2) or refused to participate in subtests during at least one testing occasion (*n* = variable). Given that our dependent variables were change scores, participants were only included in a comparison if they completed the measures at both pre- and post-dose. The NIH Toolbox was administered by clinical research coordinators whose training was overseen by clinical psychologists.

#### Dimensional change card sort test

In this test of attention and cognitive flexibility, participants are required to match a pair of pictures on one dimension (shape, color), before shifting to another dimension later in the task.

#### Flanker inhibitory control and attention test

Participants are required to attend to a central stimulus and ignore flanking stimuli.

#### Oral reading recognition test

Participants are required to read letters and words out loud.

#### Pattern comparison processing speed test

Participants must make determinations about whether two stimuli are the same or different.

#### Picture vocabulary test

Participants listen to a word and select the corresponding picture from an array of four pictures.

#### Speeded matching test

Participants are presented with a target picture along with four pictures beneath it and must select the picture that matches the target from the array.

### The repeatable battery for the assessment of neuropsychological status (RBANS)

Participants completed the List Learning subtests of the RBANS both pre- and post-dose, which is a measure of memory for a list of words that are read to them. Each list contains ten words and four attempts at recall, for a total potential score of 40. To reduce practice effects, alternate forms were used within-day and participants were limited to two testing occasions for each of the four RBANS alternate forms for the duration of the study. The RBANS was performed by clinical research coordinators whose training was overseen by clinical psychologists. To evaluate drug effects, the pre-dose score was subtracted from the post-dose score, indicating the additional number of words remembered post-dose in each condition. All eight verbal participants completed all testing occasions of the RBANS List Learning subtest, resulting in 32 change scores (8 participants × 4 dosing days) that were analyzed.

#### Clinical global impressions – improvement (CGI-I)

The CGI-I is a physician rating of improvement on a Likert scale, with a score of “1” indicating “very much improved” and a score of “7” indicating “very much worse.” A score of “4” centers the scale, indicating “no change.”

### Data analysis

#### Behavioral data

##### Intraclass correlation (ICC)

Reliability of pre-dose measures across all four visits was examined in *R* (version 4.4.0; [46]) using the *irr* package (version 0.84.1; [12]). ICC was calculated for a single measurement using a two-way random-effects model with absolute agreement [ICC(A,1)]. ICC analysis utilized change sensitive scores for NIH Toolbox Cognition Battery subtests and raw scores (total words remembered) for RBANS List Learning.

##### Linear mixed-effects models (LME)

LME were implemented using PROC MIXED in SAS® version 9.4 (SAS Institute Inc., Cary, NC) to compare outcomes between placebo and gaboxadol. Subject was included as a random effect, and treatment was modeled as a fixed effect. To examine the impact of assessment location (clinic vs. home) models initially included location and an interaction between treatment and location as predictors, which were removed if the terms were not significant. *P*-values < 0.05 were considered significant. Effects with p-values between 0.05 and 0.10 are reported as non-significant trends to aid in interpretation and hypothesis generation in this small sample. Follow-up tests are presented descriptively to characterize patterns and were not used to establish statistical significance. There were no adjustments made for multiple comparisons, as this is considered a pilot study.

#### Rest

##### Resting power

Power spectral density (PSD) was estimated using MATLAB’s *spectrogram* function with a 1 s Hamming window, 50% overlap, and frequency range of 2–100 Hz. For each channel, the median power across time windows was computed for robustness [31] and converted to decibels (dB). The resulting log-transformed PSD was stored per channel for further analysis. To estimate the spectral exponent (SPEX), a log-transformed PSD was modeled with an exponential curve using the FOOOF algorithm [9, 13]. According to these authors, a flatter SPEX is associated with excitatory states while a steeper SPEX is associated with inhibitory states, which correspond to frequency domain responses of AMPA and GABA_A receptors, respectively. However, care must be used when interpreting the SPEX values, as there are other known factors, such as cable theory (low-pass filter effect) and steady-state current loop between soma and dendrites (increases power below delta band), which can be modulated by FXS as well. The fit was restricted to the 2 to 100 Hz range and used a fixed (no-knee) aperiodic model. Periodic peaks were limited to a maximum of one per spectrum, and a threshold of 2 standard deviations was applied to detect candidate peaks. The spectral exponent, defined as the slope of the aperiodic fit, was extracted from each channel for further analysis.

To examine treatment-related changes in spontaneous EEG activity, PSD data were organized into a 2 × 2 repeated-measures design with the factors Dose (pre- vs. post-dose) and Treatment (Drug vs. Placebo), resulting in four condition-specific PSD sets. A two-way repeated-measures ANOVA was performed at each channel-frequency bin, focusing on the interaction between Dose and Treatment to identify frequency- and region-specific effects of the drug. The resulting interaction-term p-values were mapped across all channels and frequencies to identify potential regions of interest for follow-up analysis. To extract the most relevant frequency bin, a significance mask was first generated by thresholding the *p*-values at *p* < 0.05. This binary mask was summed across electrodes and smoothed using a 1-Hz moving average. The frequency bin corresponding to the peak of this smoothed sum was selected for further modeling. For each subject and session, spectral power at the selected frequency was averaged across all electrodes and entered into a linear mixed-effects model with fixed effects of Dose, Treatment, and their interaction, and random slopes and intercepts grouped by subject. Subsequently, linear mixed-effects models were applied to PSD values averaged across all channels and within each predefined frequency band: delta (2 to 4 Hz), theta (4 to 8 Hz), alpha (8 to 13 Hz), beta (13 to 30 Hz), gamma (30 to 55 Hz), and high gamma (65 Hz and above). Each model included fixed effects for Dose, Treatment, and their interaction, along with random intercepts and slopes for each subject. Model fitting was performed using maximum likelihood estimation in MATLAB R2023a (*fitlme*).

A second analysis focused on post-dose sessions to assess treatment-related differences in spontaneous EEG activity across recording environments. PSD data were grouped by Treatment (Drug vs. Placebo) and Environment (lab vs. home), and a two-way repeated-measures ANOVA was performed at each channel-frequency bin with Treatment and Environment as within-subject factors. To extract a representative frequency bin for follow-up analysis, p-values from the main effect of Treatment were thresholded at *p* < 0.05 to create a binary significance mask. This mask was summed across electrodes at each frequency and smoothed using a 1-Hz smoothing, and the frequency bin with the maximum value was selected for subsequent modeling. For follow-up modeling, post-dose spectral power at the previously selected frequency bin was averaged across all electrodes. Data were grouped by Treatment (Drug vs. Placebo) and recording Environment (lab vs. home). A linear mixed-effects model was then fit to the data with fixed effects for Treatment, Environment, and their interaction, and with random intercepts and slopes specified per subject. In a separate analysis, PSD values were averaged within each predefined frequency band and across all electrodes using only post-dose sessions. For each frequency band, a linear mixed-effects model was fit with fixed effects for Treatment, Environment, and their interaction, and with random intercepts and slopes specified per subject.

##### Peak frequency

To assess drug-related changes in the frequency of spontaneous alpha oscillations, the spectral peak within the 4 to 13 Hz range was identified for each subject and electrode during pre- and post-dose sessions in the Drug condition. For each session, the frequency bin with the maximum power in the alpha range was extracted per channel and subject. Peaks located at the lowest frequency bin were treated as invalid and excluded from further analysis. Two metrics were computed. First, for each channel, the proportion of subjects with valid alpha peaks in both sessions was calculated to quantify detection consistency. Second, for each channel, the average frequency shift from pre- to post-dose was computed using only subjects with valid peaks in both sessions. This yielded a topographic map of alpha peak frequency shifts potentially attributable to the drug. To obtain a concise descriptive summary, we selected one representative scalp electrode using a composite score defined as the product of detection consistency and the mean frequency shift. The electrode with the largest absolute score was retained, favoring it with both robust peak detection and large pre-post shifts. Peak frequency values from subjects with valid peaks in both sessions were then extracted from this electrode and analyzed with a linear mixed-effects model including dose as a fixed effect and subject-specific random intercepts and slopes. This electrode-based analysis was intended as a compact summary of the topographic findings rather than an independent basis for electrode selection and inference.

##### SPEX

To examine treatment-related changes in the aperiodic component of the EEG power spectrum, SPEX values were extracted per subject and channel for each condition: pre-dose Placebo, post-dose Placebo, pre-dose Drug, and post-dose Drug. A linear mixed-effects model was applied separately at each electrode to test for the interaction between Dose (Pre vs. Post) and Treatment (Placebo vs. Drug). The model included fixed effects for Dose, Treatment, and their interaction, and random intercepts and slopes by subject. The resulting interaction terms were used to assess the spatial distribution of drug-related effects on SPEX. A second analysis focused on post-dose sessions to evaluate whether SPEX differed between Drug and Placebo across recording environments. For each channel, a linear mixed-effects model was fit with fixed effects for Treatment (Drug vs. Placebo), Environment (lab vs. home), and their interaction, and with random intercepts and slopes by subject. Statistical terms corresponding to the main effect of Treatment and the Treatment × Environment interaction were extracted per channel.

##### ICC

To assess the test–retest reliability of our measures, we computed ICC using the ICC(A,1) formulation, which estimates absolute agreement for single measurements, for resting power spectra and SPEX. Analyses were restricted to pre-dose sessions. Three comparisons were performed: Lab-Lab, Home-Home, and Lab-Home. For resting power, at each electrode and frequency, PSD values from the two sessions were paired within subjects, and ICCs were computed across subjects. To summarize spectral reliability, ICC values were averaged across electrodes at each frequency. Scalp topographies were generated for the frequencies with the highest and lowest mean ICC values in each comparison. In a separate analysis, PSDs were first averaged within canonical frequency bands (delta: 2 to 4 Hz, theta: 4 to 8 Hz, alpha: 8 to 13 Hz, beta: 13 to 30 Hz, gamma: 30 to 55 Hz, and high gamma: 65 Hz and above), and ICCs were computed on these band-averaged values. For SPEX, values were extracted at each electrode and paired within subjects. The resulting ICC values were mapped across the scalp to assess the reliability of SPEX across repeated measures in different environments.

#### Chirp

##### Evoked activity

Epochs were extracted from −1000 ms to + 3000 ms relative to the onset of the chirp stimulus (t = 0). Event-related potentials (ERPs), event-related spectral perturbations (ERSPs), and inter-trial coherence (ITC) were computed for each epoch. For ERSP analysis, baseline correction was performed using the pre-stimulus interval from −1000 ms to 0 ms. We also computed event-related spatio-spectral perturbations (ERSSP) to quantify the spatiotemporal extent of stimulus-evoked activity, with a particular focus on potential extralemniscal involvement [19, 20], under review). For each subject, the median power and ITC values were computed across trials at each time–frequency bin. For each frequency and channel, a normal distribution was defined using the mean and standard deviation of the baseline period (−1000 to 0 ms). ERSP values falling above the 97.5th percentile or below the 2.5th percentile of this distribution were marked as significant, corresponding to event-related synchronization (ERS) or desynchronization (ERD), respectively. For ITC, only bins in the top 5% were considered significant. These binary significance masks were then summed across channels, yielding per-subject time–frequency matrices that reflect the number of significant channels at each bin. These matrices were used to characterize the spatiotemporal distribution of stimulus-related activity in both power and phase domains. To quantify stimulus-related spatiotemporal dynamics, we defined a region of interest (ROI) in the ERSSP matrix spanning 0–500 ms and 2–13 Hz. This window was selected to capture early low-frequency activity, including theta and alpha band responses. For each subject, ERSSP values within this ROI were averaged to yield a single summary measure per condition. These values were used for subsequent statistical comparisons. A linear mixed-effects model was used to analyze the ROI-averaged EEG measure. The model included fixed effects for dose, treatment group, and their interaction. A random intercept was included for each subject to account for repeated measurements.

To assess group differences in ERP amplitude at Cz, independent-samples t-tests were performed at each time point between the two experimental groups, separately for pre-dose and post-dose sessions. A two-tailed t-threshold corresponding to α = 0.05 was calculated using the median degrees of freedom from the pre-dose comparison. One subject was excluded from the pre-dose session of one group due to missing data.

##### ICC

We assessed the test–retest reliability of multiple EEG-derived measures across repeated pre-dose sessions using ICCs. Three comparisons were performed: (1) Lab-Lab, (2) Home-Home, and (3) Lab-Home. The measures included: (1) the N1-P1 peak-to-peak amplitude at Cz, (2) ERSSP-ERS values averaged within a predefined time–frequency region of interest (ROI; 0–500 ms, 2–13 Hz) corresponding to the vertex potential (VP), (3) ERSSP-ITC values within the same ROI, and (4) ERSSP-ITC values at the frequency and latency corresponding to the expected auditory steady-state response (ASSR). For the ERP analysis, the waveform was segmented to a 0–300 ms post-stimulus window, corresponding to the typical latency range of the N1-P1 complex. The peak-to-peak amplitude was calculated as the difference between the maximum and minimum voltage within this interval. Subjects with missing or invalid data were excluded pairwise for each comparison. To define the ROI for ASSR-related ITC, binary significance maps (significant = 1, non-significant = 0) were computed for ERSSP-ITC across four conditions (pre- and post-dose sessions for both treatment groups). Subject-level maps were averaged within each condition, then across conditions, yielding a group-level map reflecting the proportion of subjects showing significant ITC at each time–frequency bin. This map was summed across channels to generate a 40 Hz time series, and the time point with the highest value was selected. The resulting time–frequency bin (40 Hz at peak latency) was used to extract ERSSP-ITC values for each subject and session. For all ERSSP-based metrics, ROI values were calculated as the average number of significant channels within the specified bins. ICCs were computed using the ICC(A,1) formulation, which estimates absolute agreement for single measurements. This method was implemented in MATLAB and applied separately to each of the three comparisons using pre-dose data only.

## Results

### Demographics

**Fig. 1:**
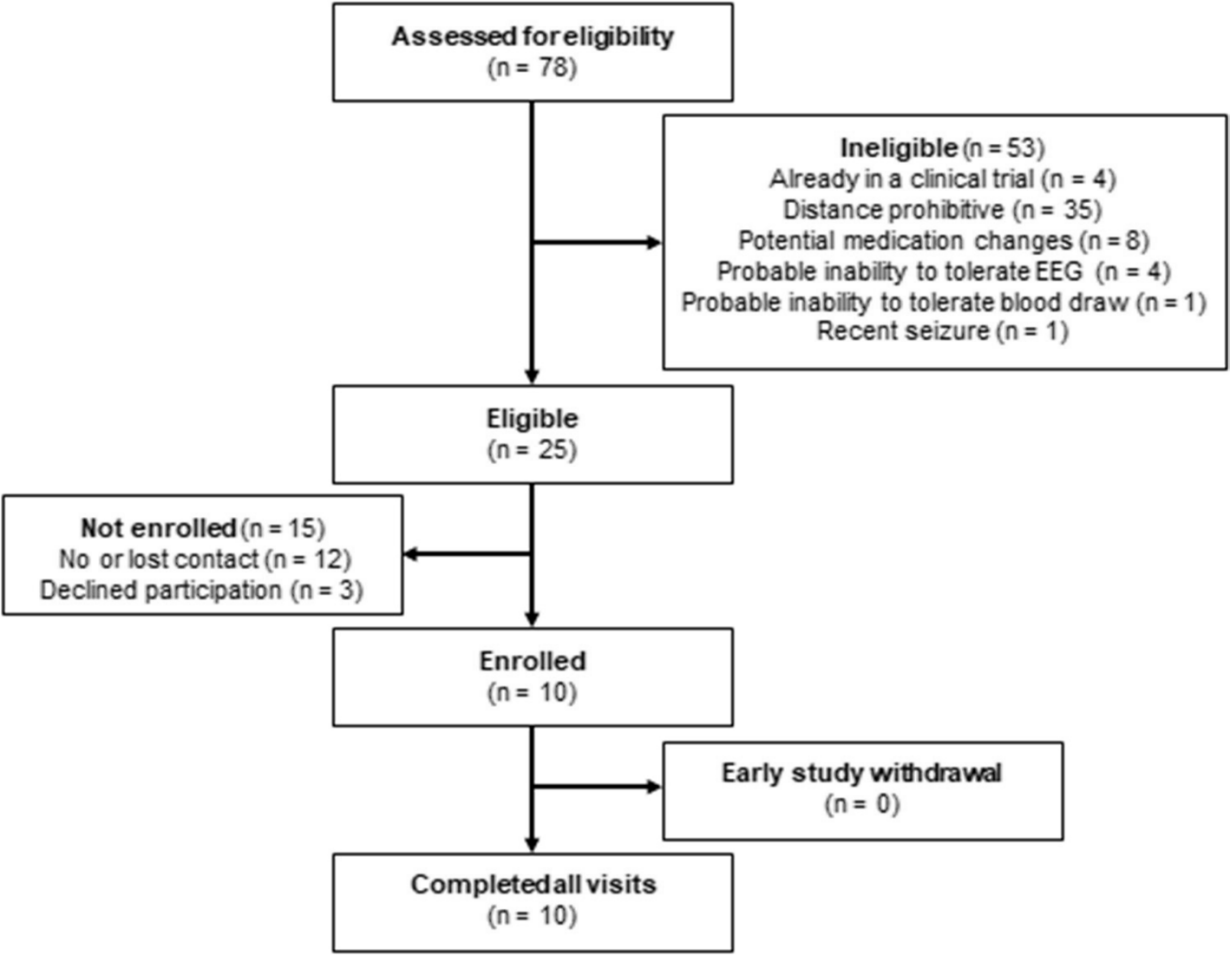
Consort diagram displaying the flow of recruitment and enrollment. Participants were recruited via an examination of historical clinic records

Ten individuals completed all four dosing visits; there was no early withdrawal from the study. See Fig. 1 for a consort diagram detailing recruitment and eligibility. Three participants did not use any relevant concomitant medications during the study, while the remaining 7 participants used at least one medication. Demographics, baseline characteristics, and concomitant medications can be found in Table 1. IQ is provided as Deviation IQ, a conversion of raw scores that allows for variability at the lower end of the scale and is believed to provide a more accurate view of intellectual functioning in individuals with neurodevelopmental disorders [51].

**Table 1:** Demographic Information and Baseline Characteristics

*Measure*	*M (SD) or % (N)*
N	10
Age (years)	29.92 (6.95)
% male	100% (10)
Race	
White	100% (10)
Ethnicity	
Hispanic or Latino	20% (2)
Not Hispanic or Latino	80% (8)
Aberrant Behavior Checklist (ABC-FXS)	
Subscale 1: Irritability/Aggression	14.00 (10.60)
Subscale 2: Lethargy	5.80 (4.42)
Subscale 3: Stereotypy	5.50 (3.57)
Subscale 4: Hyperactivity	7.60 (5.82)
Subscale 5: Inappropriate Speech	4.70 (2.63)
Subscale 6: Social Avoidance	3.40 (3.72)
Clinical Global Impressions—Severity	3.80 (0.63)
Stanford Binet-5 (SB-5)	
Deviation IQ (Abbreviated)	24.16 (22.73)
Concomitant Medications	
No psychiatric medications	30% (3)
Methylphenidate	10% (1)
2nd generation antipsychotics	60% (6)
SSRI or SNRIs	30% (3)
Valproic acid	10% (1)
Carbamazepine	10% (1)
Clomipramine	10% (1)
Atomexitine	10% (1)

*N* sample size, *M* mean, *SD* standard deviation, *IQ* intelligence quotient

Three participants in the placebo condition reported adverse events (AE), including elevated heart rate, fatigue, and emotional disturbance (anxiety, aggression, crying). No participants receiving placebo in-home reported AE. In the laboratory, four participants receiving gaboxadol reported fatigue. An additional one participant reported fever that was deemed unrelated. In-home, two participants receiving gaboxadol reported fatigue.

### Behavior

#### ICC

ICC estimates can be found in Table 2, along with previously published estimates of ICC for these measures in FXS [2, 53]. Note the variable *n* for each comparison, which reflects the number of individuals who completed pre-dose assessments in that condition and whose behavior could thus be examined for reliability. Our ICC estimates for “Across All Visits” included every person with full data (e.g., completed all pre-dose assessments); “Mean Home – Mean Lab” included individuals with at least one of each lab/home assessments; and our “Lab – Lab” and “Home – Home” required that participants complete both pre-dose assessments for that condition.

**Table 2:** Comparison of Intraclass Correlation Coefficients (ICC) Across Study Visits

	Across All Visits		Mean Home – Mean Lab		Lab – Lab		Home—Home		Shields et al. [53]		Berry-Kravis et al. [2]	
	*n*	ICC (95% CI)	*n*	ICC (95% CI)	*n*	ICC (95% CI)	*n*	ICC (95% CI)	*n*	ICC (95% CI)	*n*	ICC (95% CI)
DCCS	9	0.62 (0.30–0.88)	9	0.79 (0.31–0.95)	9	0.50 (−0.09–0.89)	9	0.62 (−0.05–0.90)	23	0.41 (0.01–0.69)	-	-
Flanker	9	0.68 (0.39–0.90)	9	0.82 (0.38–0.96)	9	0.71 (0.05–0.93)	9	0.63 (0.04–0.90)	37	0.84 (0.70–0.91)	-	-
Oral Reading	7	0.76 (0.46–0.94)	9	0.93 (0.73–0.99)	8	0.95 (0.69–0.99)	7	0.95 (0.78–0.99)	56	0.96 (0.93–0.98)	-	-
Pattern Comparison	8	0.78 (0.45–0.94)	9	0.90 (0.44–0.98)	8	0.69 (0.04–0.93)	9	0.84 (0.04–0.97)	40	0.71 (0.50–0.84)	-	-
Picture Vocabulary	9	0.94 (0.85–0.98)	10	0.98 (0.93–1.00)	9	0.91 (0.58–0.98)	10	0.97 (0.88–0.99)	57	0.79 (0.66–0.87)	-	-
Speeded Matching	9	0.86 (0.67–0.96)	9	0.92 (0.69–0.98)	9	0.88 (0.58–0.97)	9	0.86 (0.50–0.97)	-	-	-	-
RBANS	8	0.86 (0.60–0.97)	8	0.94 (0.69–0.99)	8	0.76 (−0.02–0.95)	8	0.93 (0.72–0.99)	-	-	41	0.70

In general, comparable ICC values were found across pre-dose behavioral measures in our sample, despite lower sample sizes than those found in the literature. Notably, [53] contains varying sample sizes for each measure, as they completed a data validation step prior to including data in the ICC analysis, while our data includes anyone for whom a score could be generated via the application across all testing occasions. While this adds an element of noise to our data, it reduces subjectivity associated with examiner determinations of validity. We found ICC for behavior measures to be relatively in line with the estimates in the literature, particularly with regard to Oral Reading and Pattern Comparison. Similar to Shields et al. [53], ICC was lowest for the DCCS, both within and across environments. Our Picture Vocabulary ICC was higher than Shields et al. [53], while our Flanker ICC was lower, though this effect did not differ between Lab-Lab and Home-Home Our RBANS ICC was higher within Home-Home than Lab-Lab, but both measures were at least as high as found in Berry-Kravis et al. [2].

#### LME

**Fig. 2:**
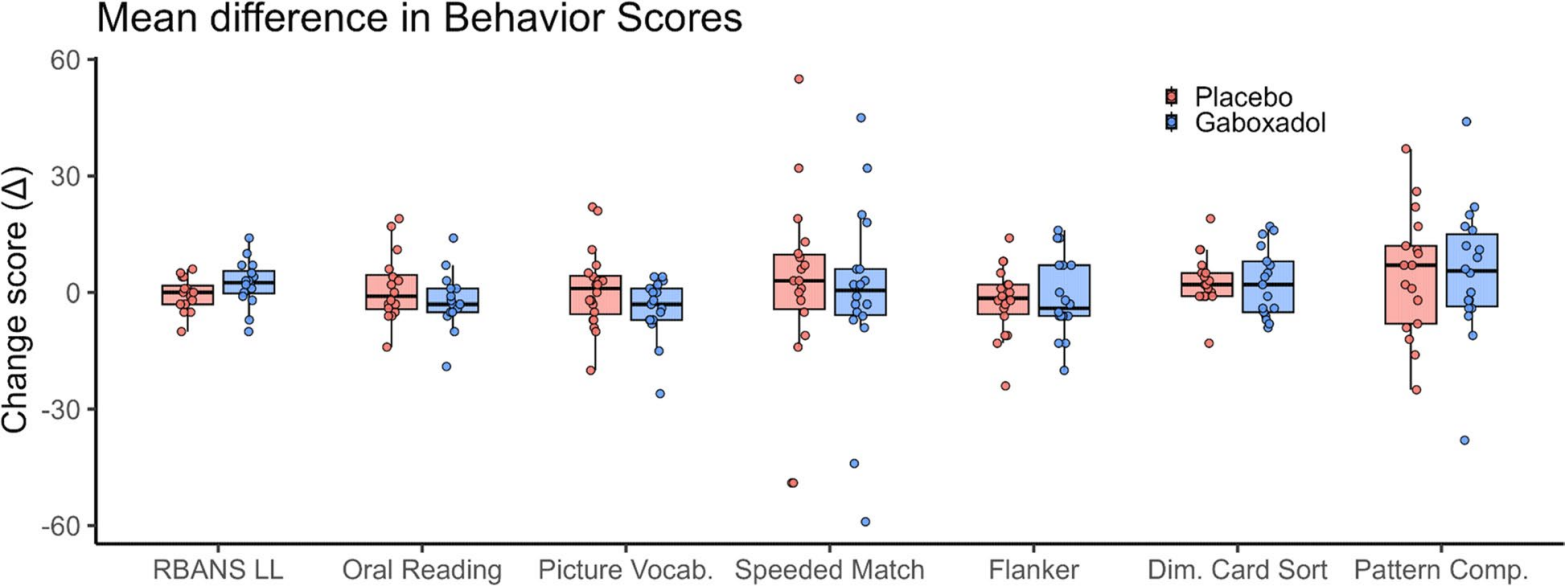
Distribution of pre- to post-dose change scores on each NIH Toolbox subtest as well as RBANS List Learning, for placebo (red) and gaboxadol (blue). The box spans the interquartile range (IQR), the horizontal bar marks the median, and the whiskers extend to 1.5 × IQR, with overlaid points for each individual at each location. Positive values reflect improvement relative to pre-dose. RBANS LL, Repeatable Battery for the Assessment of Neuropsychiatric Status List Learning subtest; NIH Toolbox measures: Oral Reading, Oral Reading Recognition Test; Picture Vocab, Picture Vocabulary Test; Speeded Match, Speeded Matching Test; Flanker, Flanker Inhibitory Control and Attention Test; Dim. Card Sort, Dimensional Change Card Sort Test; Pattern Comp., Pattern Comparison Test

Boxplots reflecting behavioral change (post- minus pre-dose, averaged across location) for each subtest can be found in Fig. 2.

#### RBANS list learning

Thirty two change scores were analyzed; all eight verbal participants completed all RBANS testing. There were no significant effects of location or interaction between treatment and location, thus these factors were removed from the model. Examining treatment alone, with each dose considered a replicate, there was a non-significant trend toward greater within-day change following gaboxadol relative to placebo (*F*(1, 23) = 3.36, *p* = 0.08, gaboxadol *M* = 2.38, placebo *M* = −0.50). However, this trend did not reach statistical significance and should not necessarily be interpreted as evidence of a treatment effect.

To characterize this pattern, a follow-up model including treatment and dose (pre/post) was conducted. This exploratory analysis showed a main effect of treatment, with higher scores in the gaboxadol condition overall regardless of pre/post dose, (*F*(1,53) = 4.50, *p* = 0.04), but no interaction between treatment and dose, (*F*(1,53) = 2.37, *p* = 0.13). Uncorrected post-hoc comparisons found that post-dose gaboxadol scores were higher than pre- and post-dose placebo, but only marginally higher than pre-dose gaboxadol. These findings should be interpreted cautiously, as our primary analysis did not reach significance and the follow-up analyses were exploratory.

#### Speeded matching test

Thirty six change scores were analyzed; one individual did not complete this test at all testing occasions. There were no main effects of treatment or location, but there was a significant interaction between treatment and location, *F*(1, 24) = 8.89, *p* < 0.01. Comparison of least squares means indicated that performance improved post-dose in placebo in-lab (*M* = 7.67) relative to placebo in-home (*M* = −4.56, *t*(24) = 2.88, *p* < 0.01) and gaboxadol in-lab (*M* = −3.00, *t*(24) = −2.51, *p* = 0.02), but not compared to gaboxadol in-home (*M* = 2.67, *t*(24) = 1.18, *p* = 0.25).

#### Flanker inhibitory control and attention test

Thirty six change scores were analyzed; one individual did not complete this test at all testing occasions. There were no main effects of treatment or location, or interaction between treatment and location (all *p*s > 0.24). With location removed from the model, there was no effect of treatment (*p* = 0.79).

#### Dimensional change card sort test

Thirty five change scores were analyzed; one individual did not complete this test at all testing occasions, and another individual did not complete the post-dose test at one occasion. There were no main effects of treatment or location, but there was a significant interaction between treatment and location, *F*(1,23.9) = 5.55, *p* = 0.03. Uncorrected post-hoc tests comparing differences of least squares means found greater within-day improvement following gaboxadol in home relative to clinic (*t*(24.4) = 2.39, *p* = 0.02, gaboxadol in-home *M* = 7.14, gaboxadol in-clinic *M* = −1.78). There was also a non-significant difference observed between gaboxadol and placebo in-clinic, *t*(23.5) = 1.75, *p* = 0.09, with larger improvements in the placebo condition than with gaboxadol (placebo in-clinic *M* = 4.56, gaboxadol in-clinic *M* = −1.78).

#### Pattern comparison processing speed test

Thirty five change scores were analyzed; one individual did not complete this test at all testing occasions, and another individual did not complete the post-dose test at one occasion. There were no main effects of treatment or location, or interaction between treatment and location (all *p*s > 0.47). With the effect of location removed from the model, there was still no significant effect of treatment (*p* = 0.88).

#### Oral reading recognition test

Thirty one change scores were analyzed; two nonverbal participants did not complete testing at all testing occasions, and one individual did not complete pre-dose testing at one occasion. There were no main effects of treatment or location, or interaction between treatment and location (all *p*s > 0.16), and there was no effect of treatment when location was removed from the model (*p* = 0.28).

#### Picture vocabulary test

Thirty nine change scores were analyzed; one individual did not complete pre-or post-dose testing at one occasion. There was no main effect of treatment or interaction between treatment and location, and thus these factors were removed from the statistical model. Looking only at the effect of treatment, there was a non-significant trend toward lower scores following gaboxadol compared to placebo (*F*(1, 28.6) = 3.09, *p* = 0.09, gaboxadol *M* = −4.00, placebo *M* = 0.75).

To understand this further, we conducted a model utilizing treatment and dose (pre/post) as factors. Here, we found no main effect of treatment or dose, but a non-significant interaction between treatment and dose, *F*(1, 65) = 3.27, *p* = 0.08. Uncorrected post-hoc tests examining differences of least squares means found that scores in the gaboxadol condition significantly decreased between pre- and post-dose (gaboxadol pre-dose *M* = 475.8, post-dose *M* = 471.8) but did not otherwise significantly differ from scores in the placebo condition (placebo pre-dose *M* = 472.85, post-dose *M* = 473.60). These findings suggest that the apparent effect may reflect non-specific variation rather than a systematic drug-related change.

#### Clinical global impressions – improvement

All participants had ratings for all CGI-I occasions. Most dosing occasions received a physician rating of “4” for “no change,” though three occasions saw a “3” indicating that the participant had “minimally improved.” All three occasions were in the gaboxadol condition and represented three distinct participants. Two of the occasions were at in-home visits and one was in-clinic.

### EEG

#### Power spectral density (PSD) and spectral exponent (SPEX) analysis

##### The drug increases EEG power at 6 Hz

**Fig. 3:**
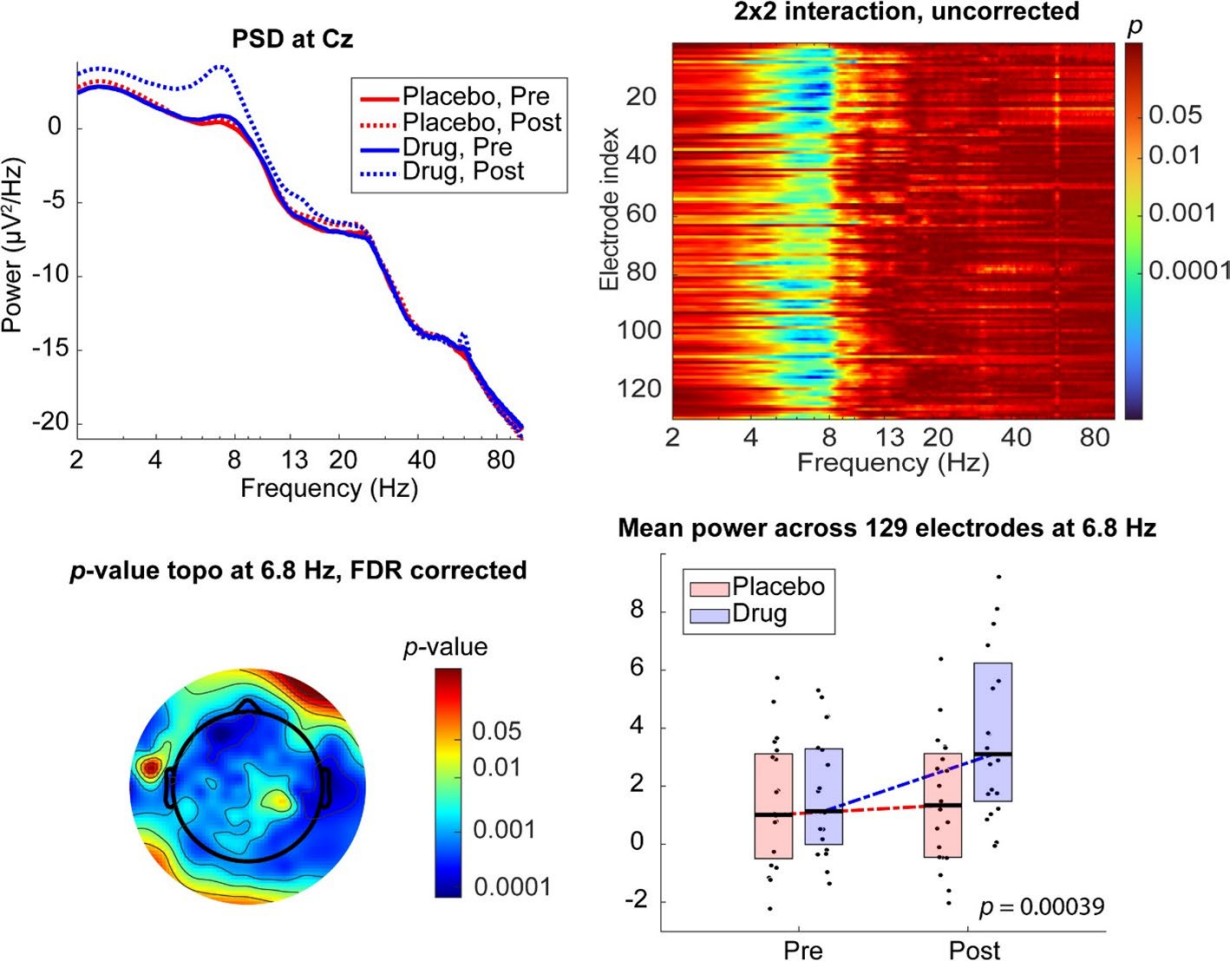
PSD analysis for Pre-Post comparison between the Placebo and Drug conditions using a 2 × 2 design. Top left: representative data recorded at Cz. Top right: results from the 2 × 2 test across 129 electrodes and 491 frequency bins. Uncorrected *p*-values are plotted. Bottom left: a scalp topography showing uncorrected *p*-values at 6.8 Hz in which the most electrodes showed significant interactions. Bottom right: results from the interaction test on mean powers across 129 electrodes at 6.8 Hz. The edges of the box plots indicate quartiles, the horizontal lines indicate median values, and the dots indicate the scalp electrode-averaged power for each individual at the first and second visits, shown separately

We calculated PSD of resting-state EEG data to compare Pre-Post conditions between the Placebo and Drug conditions, which employed a 2 × 2 factorial design. The results are shown in Fig. 3. The targeted 2 × 2 interaction showed a characteristic frequency-dependent pattern peaking in the theta band (4–8 Hz), which was confirmed across the majority of electrodes. The peak detection on electrode-averaged results identified that the peak of the statistical results was localized at 6.8 Hz, where the interaction calculated within the LME model was statistically significant, *F*(1, 76) = 13.8, *p* = 0.00039. The responder rate, which is defined as the number of datasets that showed increase in the Drug Post condition, was 80%.

**Fig. 4:**
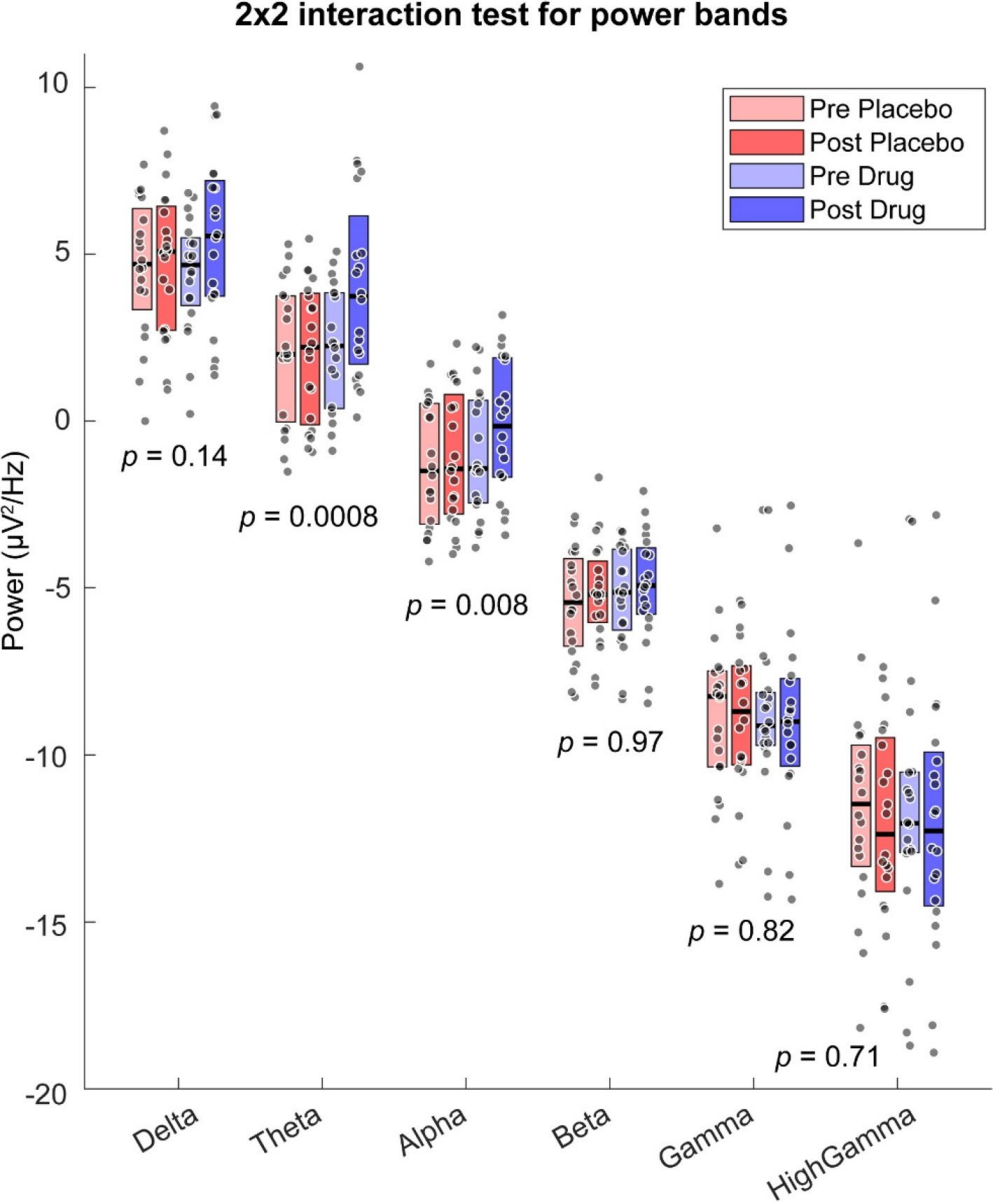
The 2 × 2 interactions tested for power bands. Both theta-band (4–8 Hz) and alpha-band (8–13 Hz) power showed significant interactions in which post-Drug condition demonstrated prominent power increase. Gray dots represent the scalp electrode-averaged power for each individual at the first and second visits, shown separately

Next, we separated these estimates into six conventional frequency bands (Delta, 1–4 Hz; Theta, 4–8 Hz; Alpha, 8–13 Hz; Beta, 13–30 Hz; Gamma, 30–55 Hz; High Gamma, 65–100 Hz) and applied the same test for the mean EEG power of each frequency bands. The results are shown in Fig. 4. A significant interaction was observed in the theta band (*F*(1,76) = 12.29, *p* = 0.0008) and the alpha band (*F*(1,76) = 7.43, *p* = 0.0080). Other frequency bands did not reach statistical significance. We conclude that the drug increases EEG power in the theta and alpha bands, with the effect centered at 6.4 Hz. The responder rates were: delta band, 85%; theta band, 80%; alpha band, 80%; beta band, 65%; gamma band, 45%; and high gamma band, 50%.

###### No significant difference between Lab and Home

**Fig. 5:**
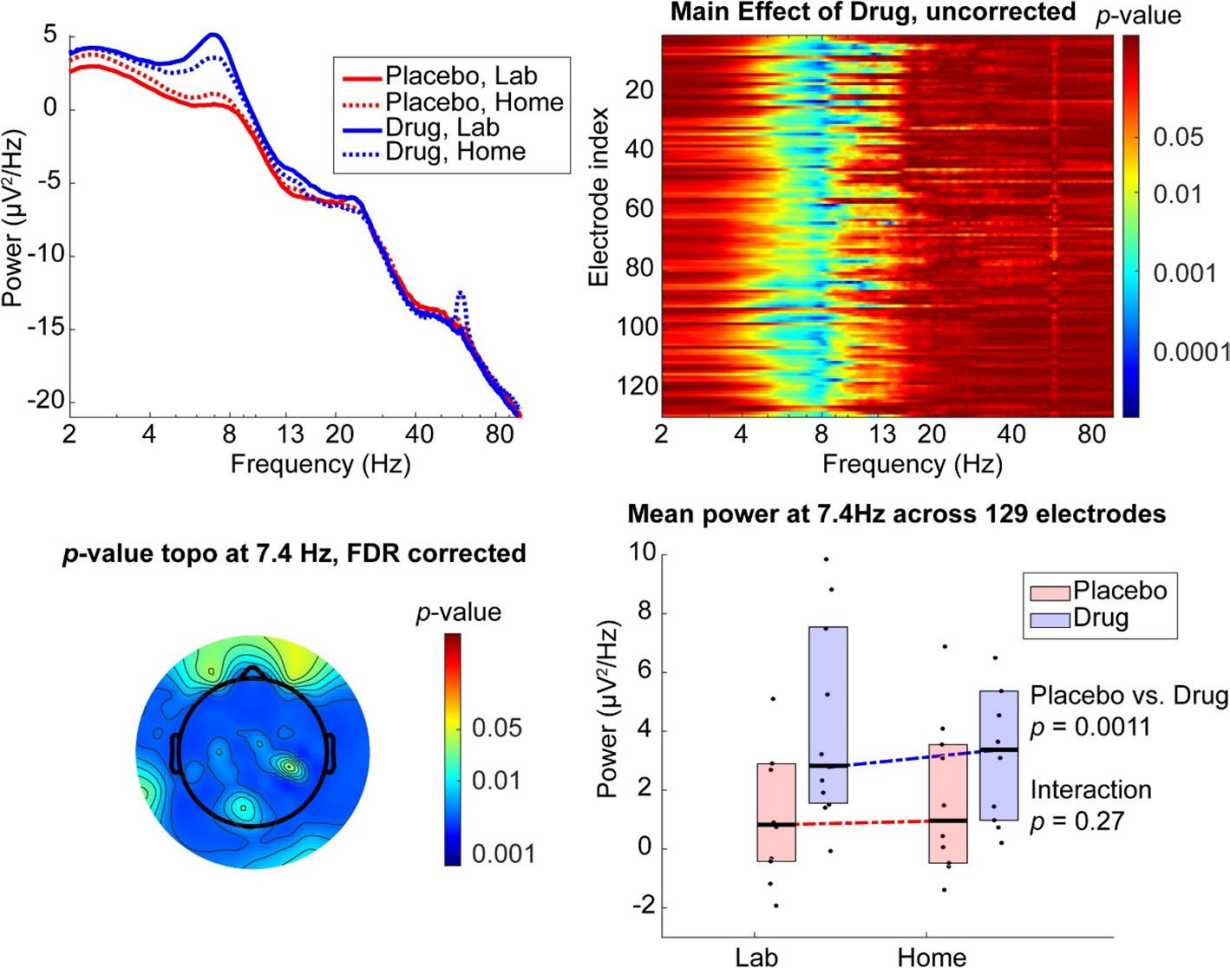
PSD analysis for post-dose Lab-Home comparison between Placebo and Drug conditions using a 2 (Lab, Home) × 2 (Placebo, Drug) design. Top left: a representative data recorded at Cz. Top right: results showing the main effect of Drug across 129 electrodes and 491 frequency bins. Uncorrected *p*-values are plotted. Neither the 2 × 2 interaction tests nor the main effect of Location showed interpretable results. Bottom left: a scalp topography of the uncorrected *p*-values at 7.4 Hz in which the most electrodes showed the significant main effect of Drug. Bottom right: results from the interaction test on mean powers across 129 electrodes at 6.4 Hz. The edges of the box plots indicate quartiles, the horizontal lines indicate median values, and the dots indicate the scalp electrode-averaged power for each individual at the first and second visits, shown separately

Based on the results confirmed on 3.3.1.1, we asked whether there was a difference between Lab and Home conditions only in the Post-dose condition. This analysis also employed 2 × 2 design, but the interaction was not significant. Instead, we observed a clear main effect of Drug > Placebo. Thus, we focus on the main effect. The results are shown in Fig. 5. Unlike Fig. 3, the electrode x frequency plot as well as the *p*-value scalp topography show the main effect of Drug. The mean power across all electrodes showed a peak statistical value at 7.4 Hz again where the main effect Drug > Placebo was significant, *F*(1, 36) = 12.7, *p* = 0.0011, while the interaction was not significant.

**Fig. 6:**
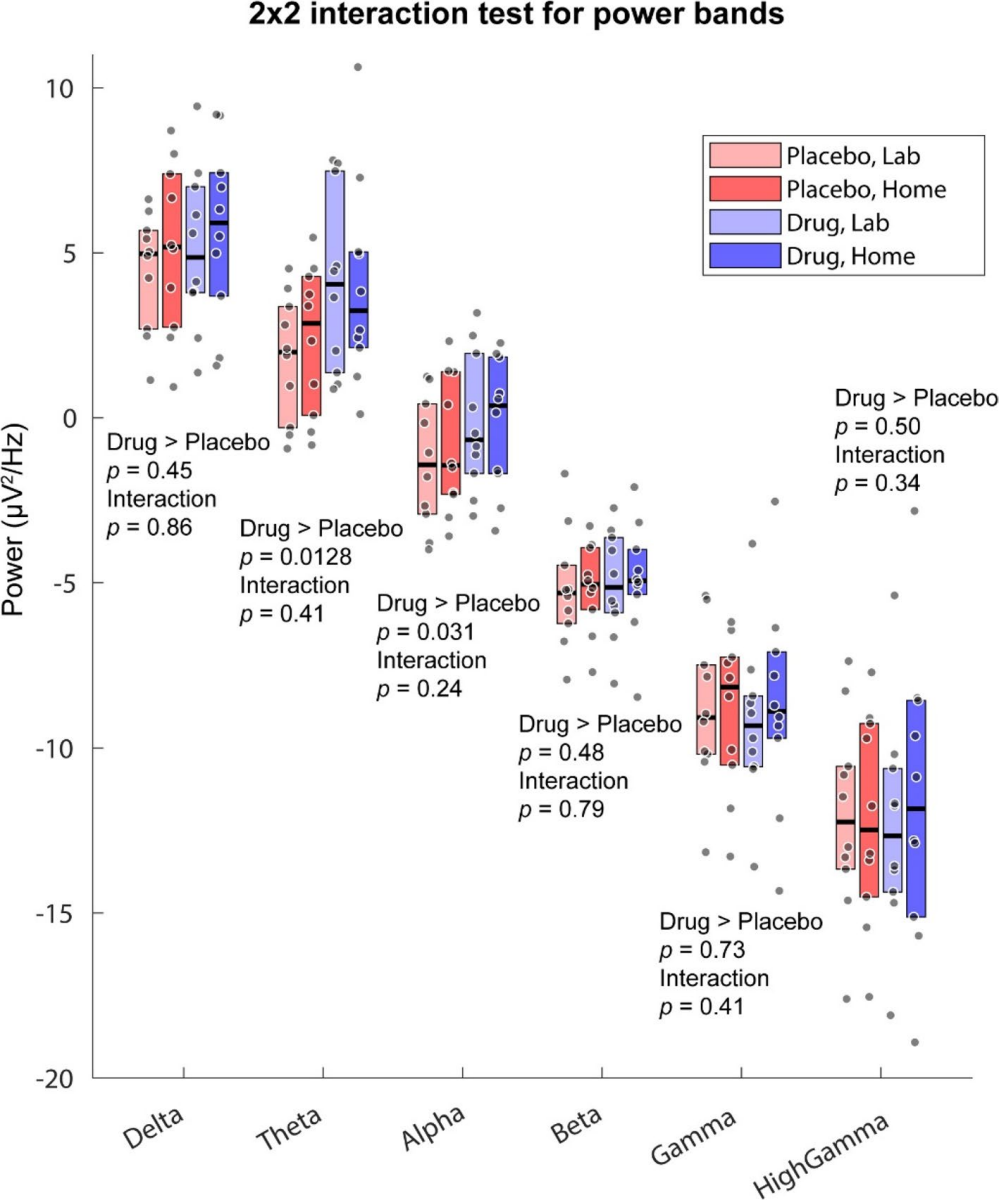
The 2 × 2 interactions tested for power bands. Both theta-band (4–8 Hz) and alpha-band (8–13 Hz) power showed a significant main effect of Drug. None of Lab vs. Home contrasts or interactions showed significant results. Gray dots represent the scalp electrode-averaged power for each individual at the first and second visits, shown separately

Next, we separated these estimates into six conventional frequency bands and applied the same test for the mean EEG power of each frequency band. The results are shown in Fig. 6. Both the theta band and alpha band showed statistical significance: *F*(1, 36) = 6.87, *p* = 0.013 and *F*(1, 36) = 5.04, *p* = 0.031, respectively. Other frequency bands did not reach statistical significance. We conclude that the Lab-Home difference was statistically insignificant; theta and alpha power augmentation was found in both environments.

###### **The drug slows alpha peak frequency**

**Fig. 7:**
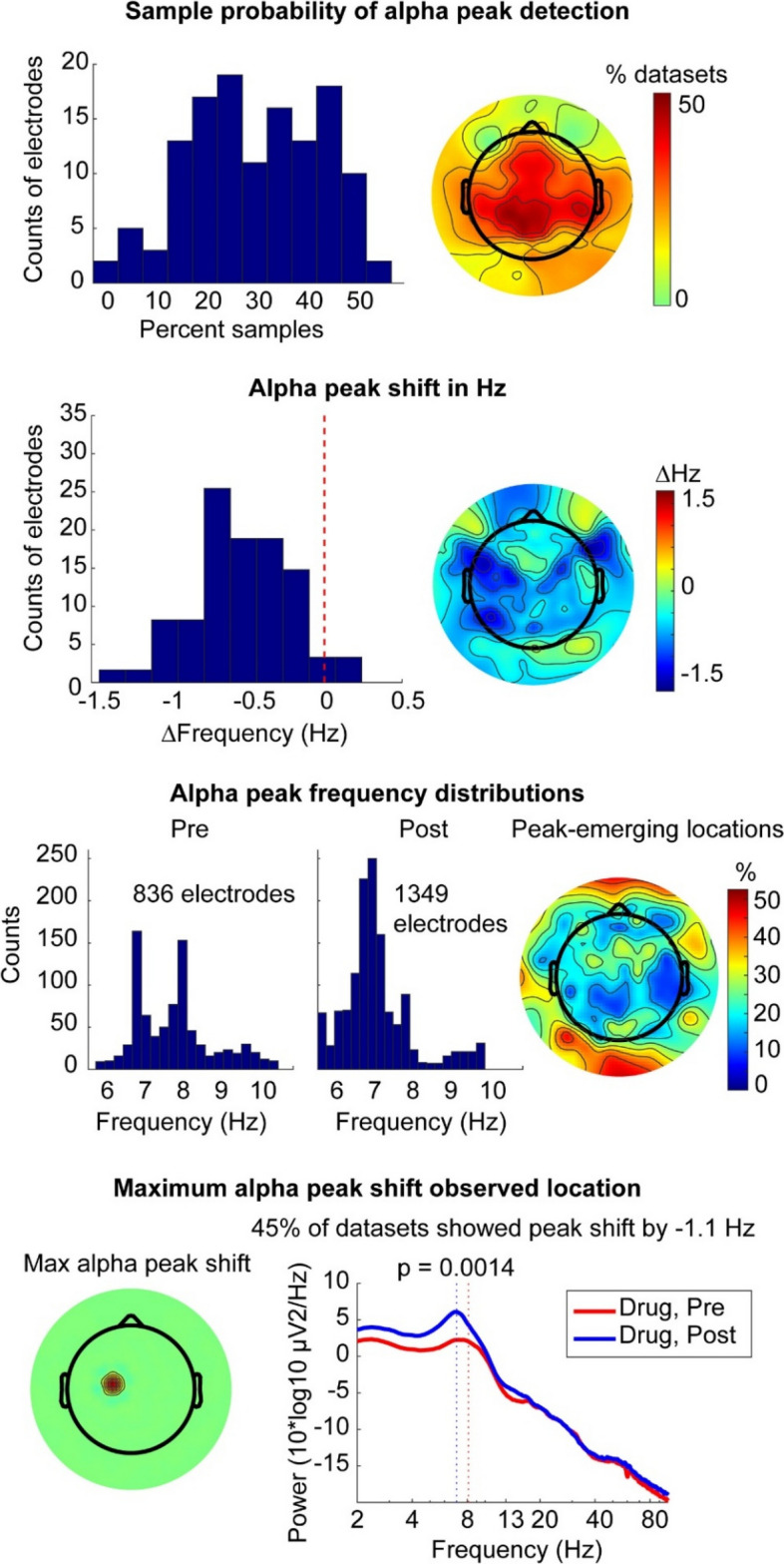
The effect of the drug on alpha peak frequency. Top left: a histogram of sample probability of alpha peak presence across electrodes. Top right: a scalp topography of the sample probability of alpha peak presence. Second row left: a histogram of the amount of alpha peak shift. Most of them show a negative shift. Second row right: a scalp topography of the alpha peak frequency shift. Third row, histograms of peak ‘alpha’ frequencies before (left) and after (right) administering the drug. Bottom left, an illustration of an electrode site that showed the maximum product of the percent datasets and the amount of the peak shift. Bottom right, PSD comparison at the selected electrode site where 45% of datasets showed a peak downshift of 1.1 Hz on average

We examined the drug's impact on alpha peak frequency by comparing data in the Drug condition Pre- and Post-dose. In conducting this analysis, we ensured not to overgeneralize the assumption that, while an alpha peak is present in most grand-mean PSD plots in general, it does not guarantee that every individual exhibits a detectable alpha peak. Thus, we first quantified the rate of peak alpha presence across subjects and electrodes. The results are shown in Fig. 7. On average, only 30.2% of datasets showed the alpha peak (SD 13.2) in a given electrode. For an additional comparison of this value with that of a resting EEG database, see Supplement S2. Next, we quantified the frequency shift for each electrode that showed a pair of alpha peaks in both Pre- and Post-dose conditions. Most electrodes showed a negative shift: on average, the peak frequency was shifted 0.53 Hz (SD 0.32) down. We evaluated histograms of the peak frequencies between Pre and Post conditions. The drug increased the number of detectable peaks from 836 to 1349, with a prominent peak around 7 Hz. This comparison also revealed that the peak frequency distribution in the Pre condition was not centered at the conventional alpha range (8–13 Hz) but the distribution is centered between 7 and 8 Hz. Finally, a representative electrode was selected that maximized the balance between the number of datasets with detectable peaks and the amount of peak frequency shift. This representative electrode analysis confirmed a significant downshift of peak frequency by 1.1 Hz.

**Fig. 8:**
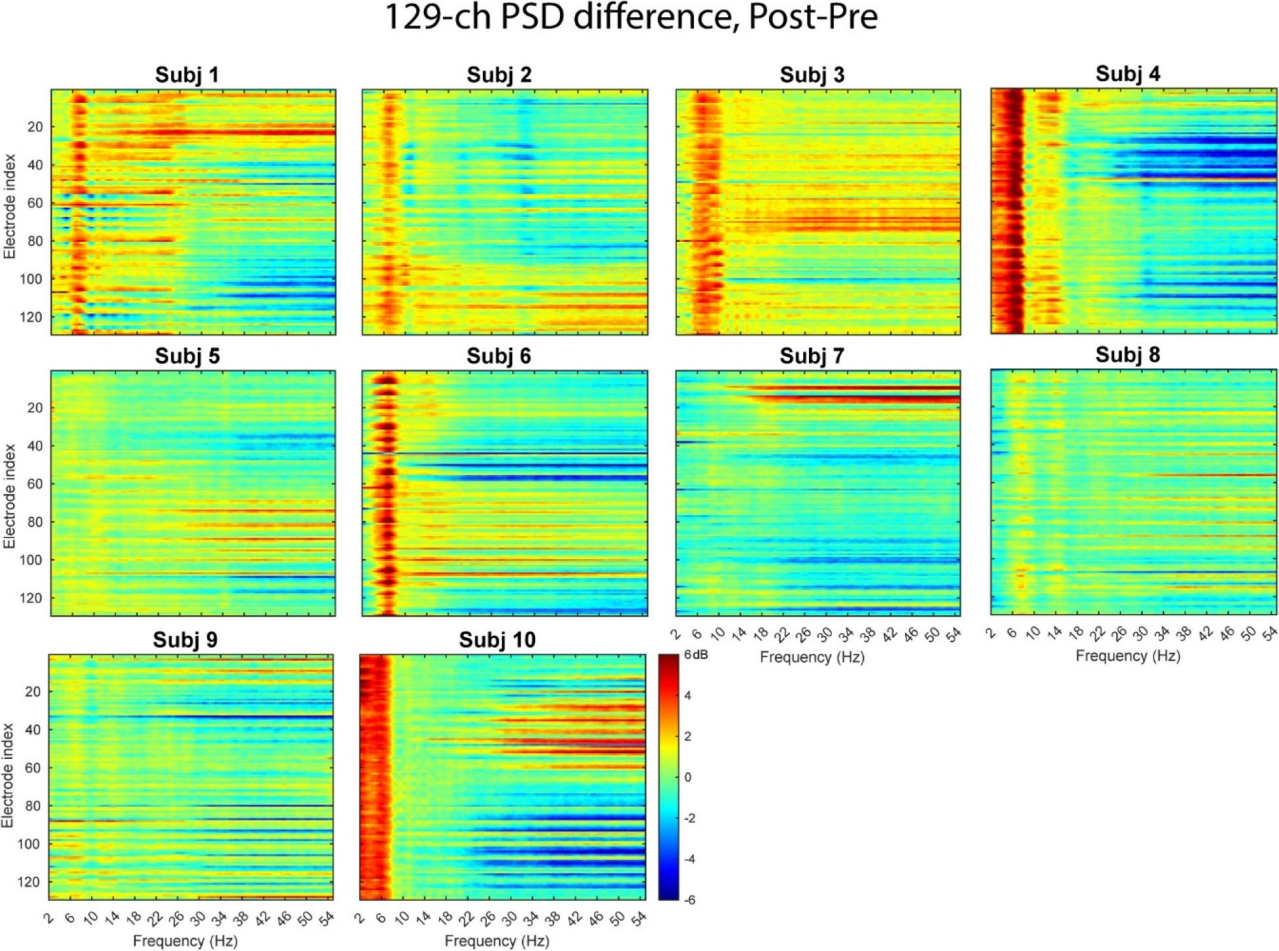
Individual difference was analyzed to determine responders and non-responders. Each plot show 129-ch broad-band PSD differences in Post–Pre that are stacked vertically. Note subject 1, 2, 3, 4, 6, 10 show prominent power increase below the alpha band, while the remaining subjects show little to no clear responses

To confirm the group-level analysis results in individual subjects, we conducted an additional analysis to evaluate individual differences in responsiveness to the drug in EEG power. We included all electrodes and frequency bins to ensure the broadest sensitivity. The result is shown in Fig. 8. Visual inspection revealed that six subjects showed clear power increase after taking the drug below the alpha band (subjects 1, 2, 3, 4, 6, 10). In contrast, the remaining subjects showed weak (subject 5, 8, 9) to no clear (subject 7) power increase. The result is consistent with the data shown in Fig. 7 that about 45% of subjects showed both (1) detectable alpha peak and (2) measurable peak shift at a given electrode site.

###### The Drug biases the E/I balance toward inhibition

**Fig. 9:**
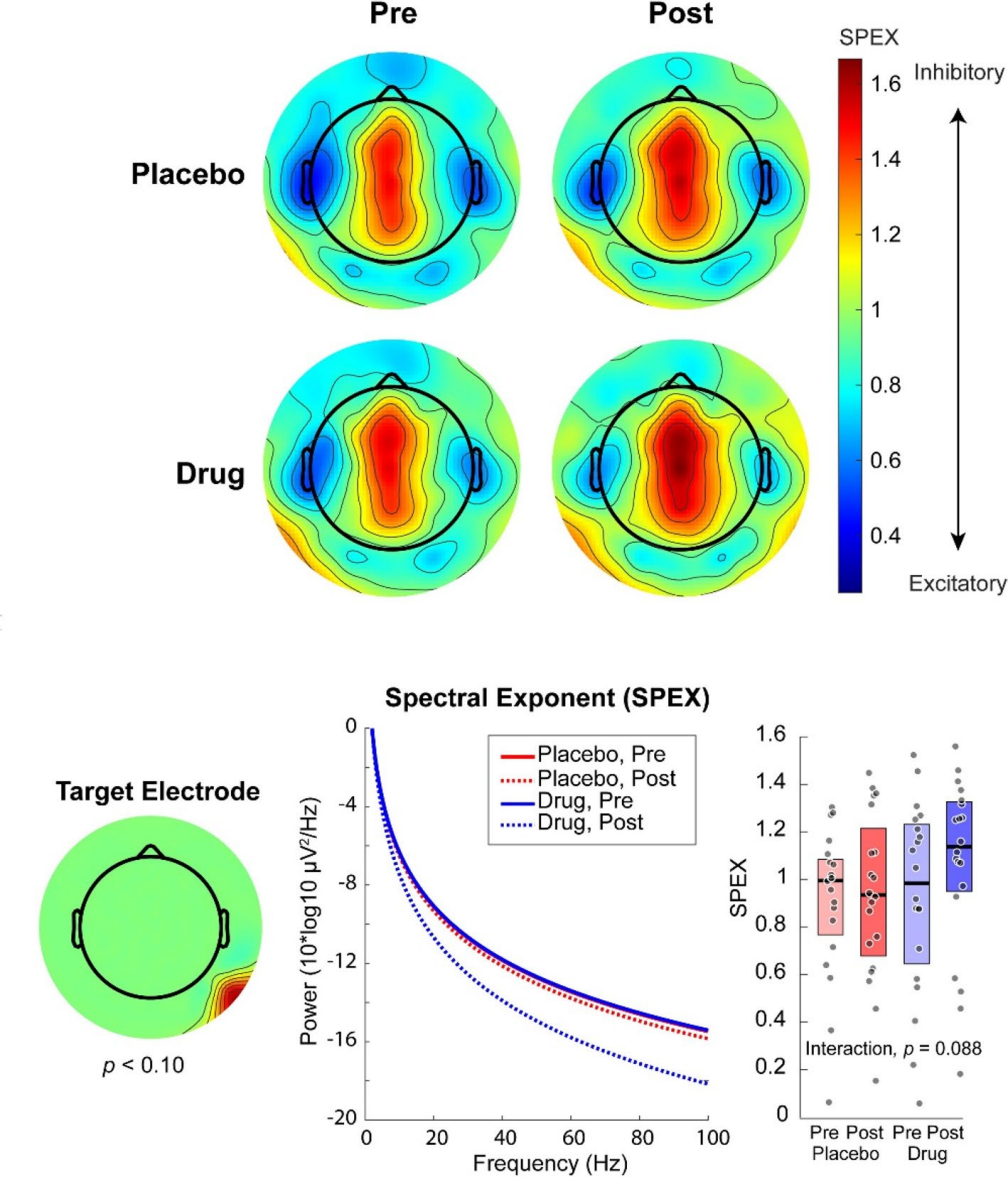
Spectral exponent (SPEX) analysis for Pre-Post comparison for Placebo and Drug conditions using a 2 × 2 design. Top, SPEX topographies for the 2 × 2 factorial design. Bottom left: a right occipito-temporal electrode location showed trends toward significanceBottom middle: Grand-mean fitted SPEX models for the midline electrode. Bottom right: grand-mean SPEX values for each condition. The edges of the box plots indicate quartiles, the horizontal lines indicate median values, and the dots indicate individual data points

We evaluated the effect of the drug on estimated E/I balance by calculating SPEX for all scalp electrodes. We compared Pre-Post conditions between the Placebo and Drug conditions, which employed a 2 × 2 factorial design. The results are shown in Fig. 9. None of the electrodes showed significant interaction. However, only one electrode showed a trend toward significance, with a SPEX coefficient increase in the Drug Post condition, a direction that would indicate a E/I balance shift toward inhibition. For the Drug condition, the responder rate was 60%. Although this finding had a limitation in statistical significance, the Drug > Placebo pattern was more consistently observed in the next analysis.

**Fig. 10:**
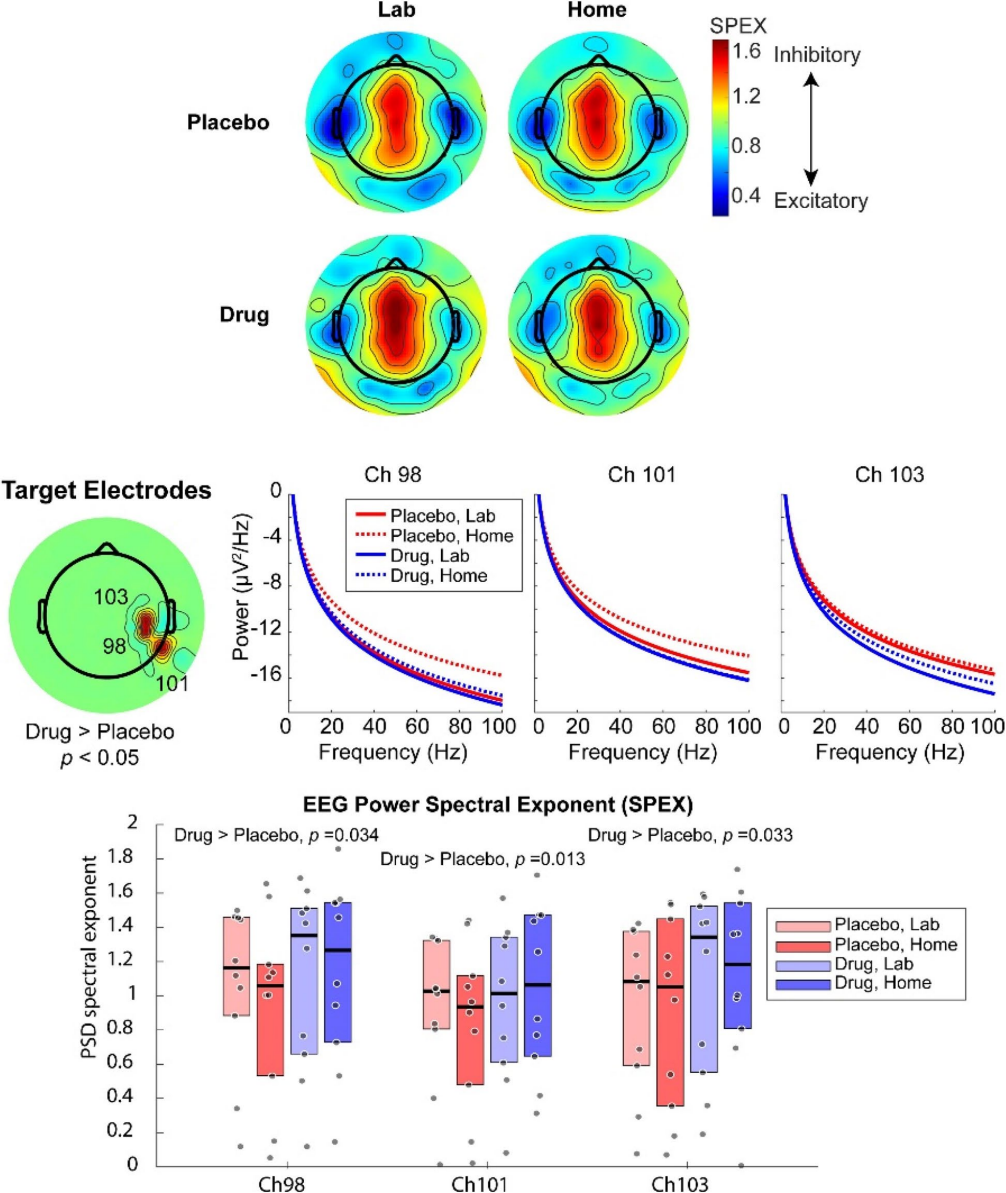
Spectral exponent (SPEX) analysis for post-dose Lab-Home comparison for Placebo and Drug conditions using a 2 (Lab, Home) × 2 (Placebo, Drug) design. Top, SPEX topographies for the 2 × 2 factorial design. Middle left: three electrodes showed the main effect of Location at uncorrected *p* < 0.05. Middle right: All three electrodes showed Drug > Placebo. Bottom: Grand-mean fitted SPEX models for electrodes showing significant main effect. The edges of the box plots indicate quartiles, the horizontal lines indicate median values, and the dots indicate individual data points

Next, we asked whether there was a difference between Lab and Home conditions for Drug and Placebo conditions, only in the Post-dose condition. Thus, this analysis employed a 2 × 2 design. The effect of the interaction reached statistical significance in some of the electrodes, which were however not interpretable. These results are reported in Supplement S3. We observed the significant main effect of Drug > Placebo at three recording sites distributed at broad parieto-temporal regions. The results are shown in Fig. 10. Notably, all three electrode sites showed a consistent pattern of Drug > Placebo, suggesting the potential for the drug to balance E/I balance toward inhibition in the right occipito-temporal area. The responder rates were as follows: Ch 98, Lab 70.0%, Home, 60.0%; Ch 101, Lab 70.0%, Home, 40.0%; Ch 103, Lab 50.0%, Home, 30.0%.

###### **Intraclass Correlation Coefficients (ICCs) between Lab and Home are comparable**

**Fig. 11:**
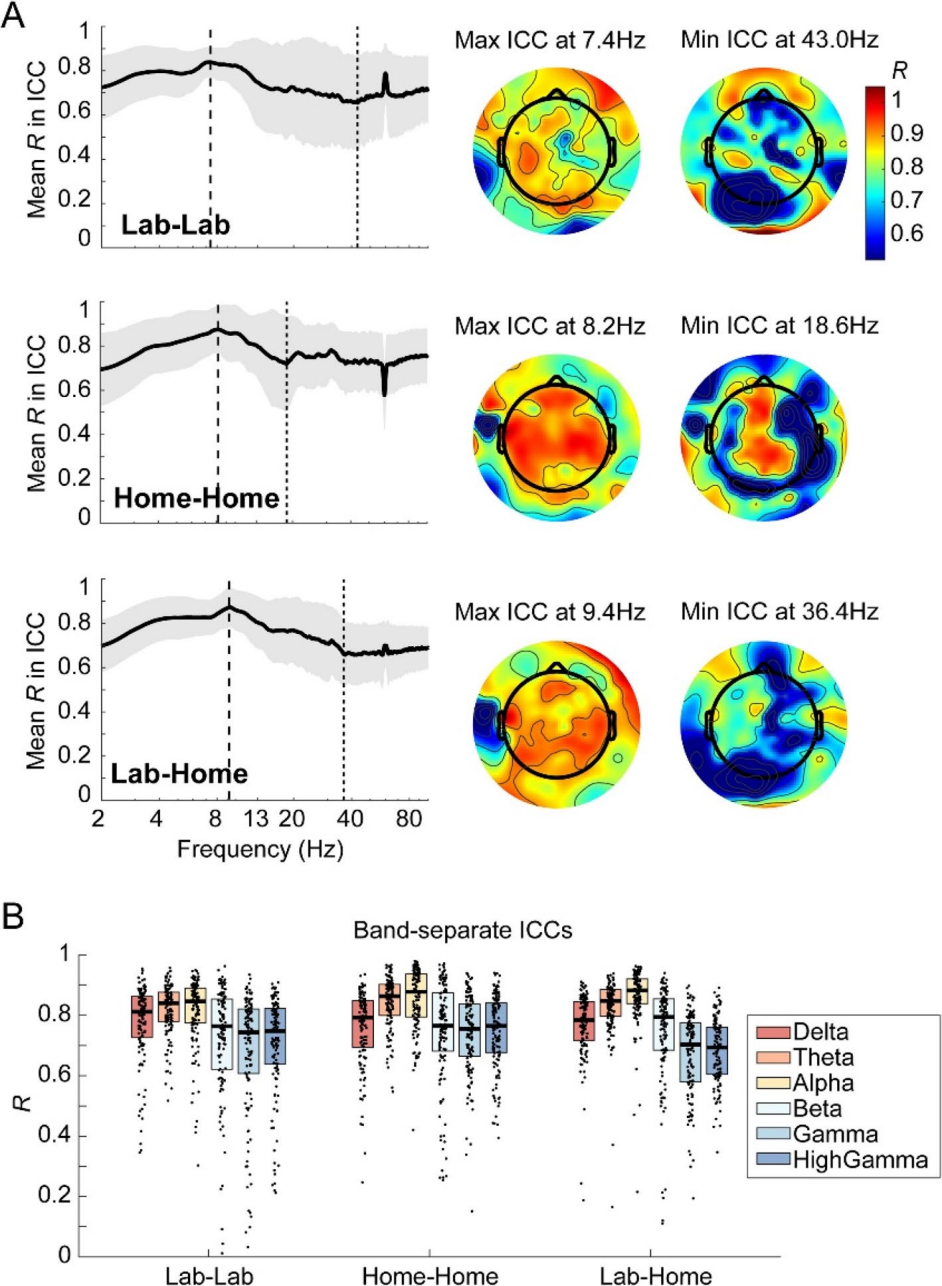
ICCs of PSDs calculated for Lab-Lab, Home-Home, and Lab-Home conditions across all the electrodes and frequency bins. A, Top left: Frequency-domain plot of average ICCs across all electrodes for the Lab-Lab comparison. A shade represents ± 1 SD across electrodes. A broken vertical line indicates a frequency where ICC was maximal, while a dotted vertical line indicates a frequency where ICC was minimal. Top right: scalp topographies of ICCs at the maximum (left) and the minimum (right) frequency. The color scale is common across all the topographies. Middle: the same plot for the Home-Home comparison. Botton: the same plot for the Lab-Home comparison. B: Band-separate ICC for three comparisons. The edges of the box plots indicate quartiles, the horizontal lines indicate median values, and the dots indicate grand-mean ICC for each electrode. Delta, 1–4 Hz; Theta, 4–8 Hz; Alpha, 8–13 Hz; Beta, 13–30 Hz; Gamma, 30–55 Hz; High Gamma, 65–100 Hz

We tested the effect of location by comparing data in the Pre-dose condition between Lab and Home. The results are shown in Fig. 11 and presented in Table 3. Across the three contrasts, ICCs were generally similar across the frequency spectrum (range 0.67–0.87). All three comparisons showed that peak ICC values were identified in the 7–10 Hz range. Visual inspection of the maximum and minimum ICC for each condition showed disparate patterns. In addition, to allow comparison with previous studies [24], we generated ICCs by frequency band across the three contrasts. We conclude that reproducibility between Lab-Home recordings is comparable to those of Lab-Lab and Home-Home.

**Table 3:** Test–Retest Reliability (ICC) of Pre-dose Resting-State PSDs by Frequency Band

**Frequency Band**	**Lab-Lab**	**Home-Home**	**Lab-Home**	Liu et al. [24]**, Mean**
Delta (2–4 Hz)	0.776 (0.127)	0.752 (0.144)	0.769 (0.108)	0.803
Theta (4–8 Hz)	0.818 (0.085)	0.837 (0.120)	0.828 (0.093)	0.771
Alpha (8–13 Hz)	0.812 (0.115)	0.847 (0.129)	0.864 (0.097)	0.852
Beta (13–30 Hz)	0.699 (0.244)	0.742 (0.173)	0.746 (0.165)	0.785
Gamma (30–55 Hz)	0.675 (0.213)	0.737 (0.134)	0.672 (0.140)	0.676
Hi-Gamma (65 + Hz)	0.703 (0.165)	0.747 (0.125)	0.683 (0.106)	

*Mean ICC across electrodes, SD in parentheses*

**Fig. 12:**
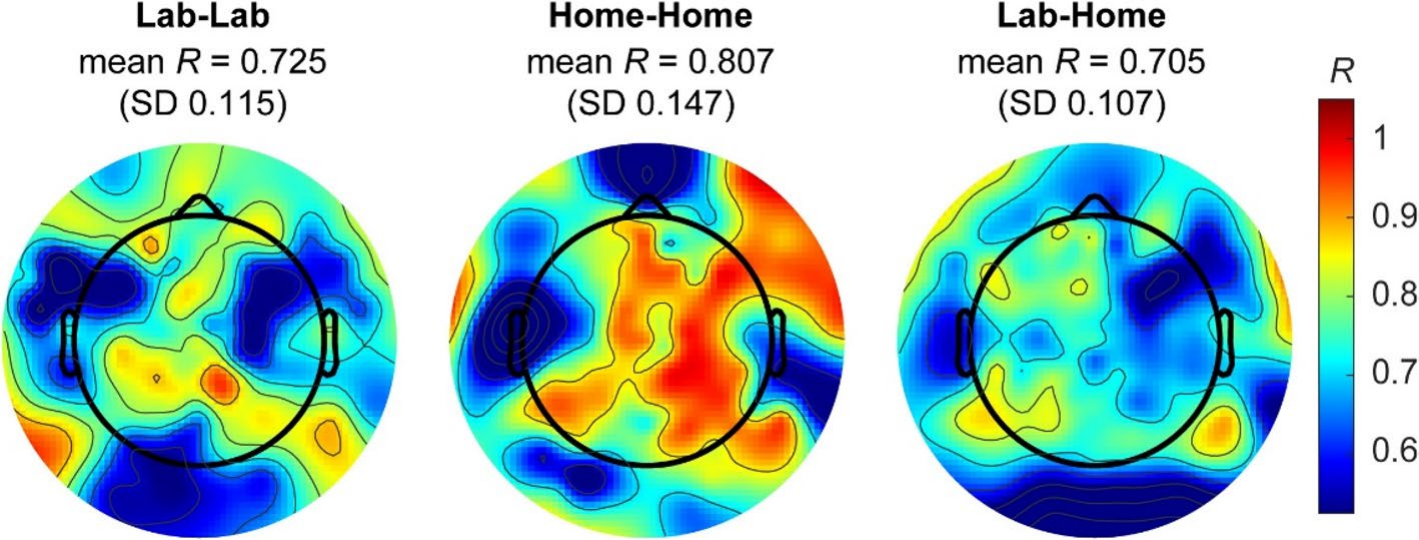
ICCs of SPEX calculated for Lab-Lab, Home-Home, and Lab-Home conditions across all the electrodes. Left: ICCs of SPEX for the Lab-Lab comparison. The mean and standard deviation values were calculated across electrodes. Middle: ICCs of SPEX for the Lab-Lab comparison. Right: ICCs of SPEX for the Lab-Home comparison

For interest, we conducted the same ICC analysis on the SPEX data. The results are shown in Fig. 12. The mean ICC values across the electrodes were in the range of 0.70–0.81, which were comparable to the ICC values from the PSD test. Visual inspection did not confirm a similar topographical pattern across locations.

#### Auditory-evoked event-related potential (ERP) analysis in time and time–frequency domains

##### Vertex potential (VP) is insensitive to the drug effect

**Fig. 13:**
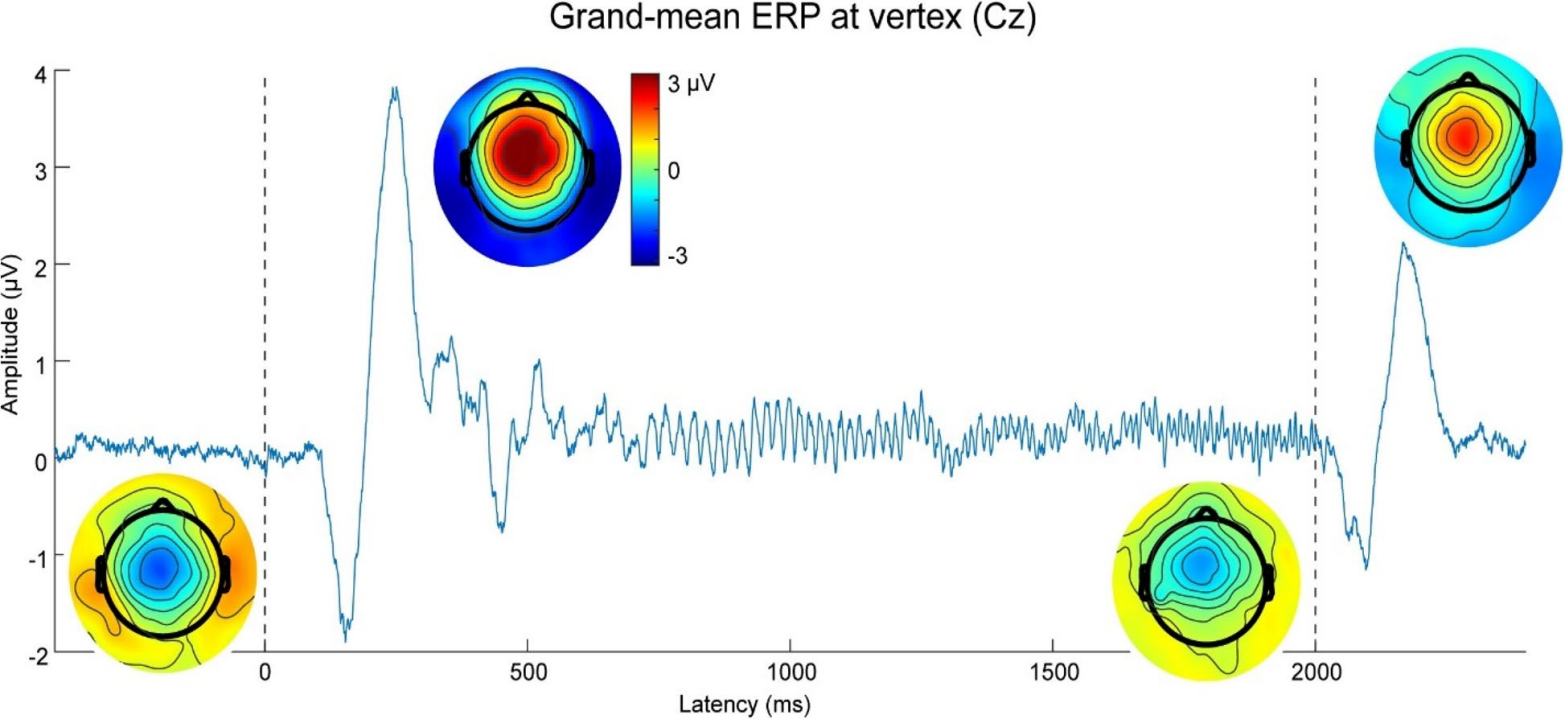
Grand-mean auditory-evoked ERP recorded at vertex (Cz). Onset and offset of the auditory chirp stimuli are indicated by broken lines at 0 and 2000 ms, respectively. Onset- and offset-evoked vertex potentials (VPs) are present. For each negative and positive peaks (N1 and P1, respectively), scalp topographies of potentials are plotted. All scalp topographies share the same color scale

To start the auditory-evoked ERP analysis, we first evaluated a large-amplitude phenomenon with a transient response to the onset and offset of the auditory stimuli that showed a central scalp distribution. The results are shown in Fig. 13. We identified this evoked response as a classical VP that consists of biphasic N1-P1 components. Between the onset- and offset-evoked VPs, we can observe the oscillatory waves in response to the chirp-modulated stimulus with a plateau between 500 and 2000 ms.

**Fig. 14:**
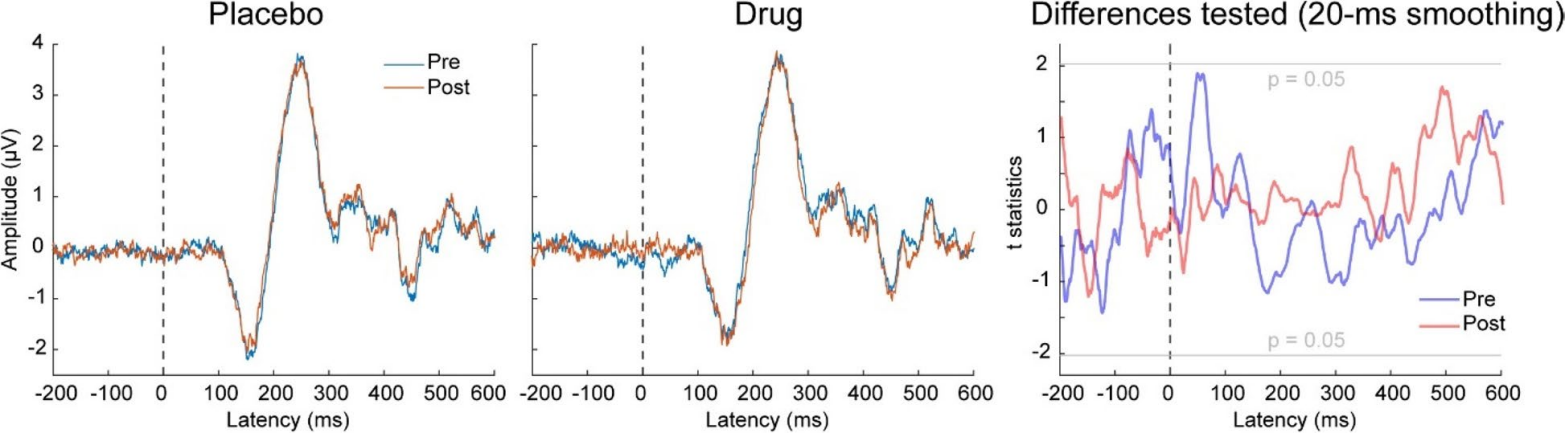
Detail view of the onset-evoked VPs at Cz for Pre-Post comparison between the Placebo and Drug conditions using a 2 × 2 design

In the initial time-domain analysis, we targeted the onset-evoked VP. We compared the effect of Pre-Post conditions between the Placebo and Drug conditions on the VP, which employed a 2 × 2 factorial design. The results are shown in Fig. 14. The difference between Pre and Post did not reach statistical significance. We conclude that the drug did not affect VP amplitudes.

##### **Power modulations on VP are insensitive to the drug effect**

**Fig. 15:**
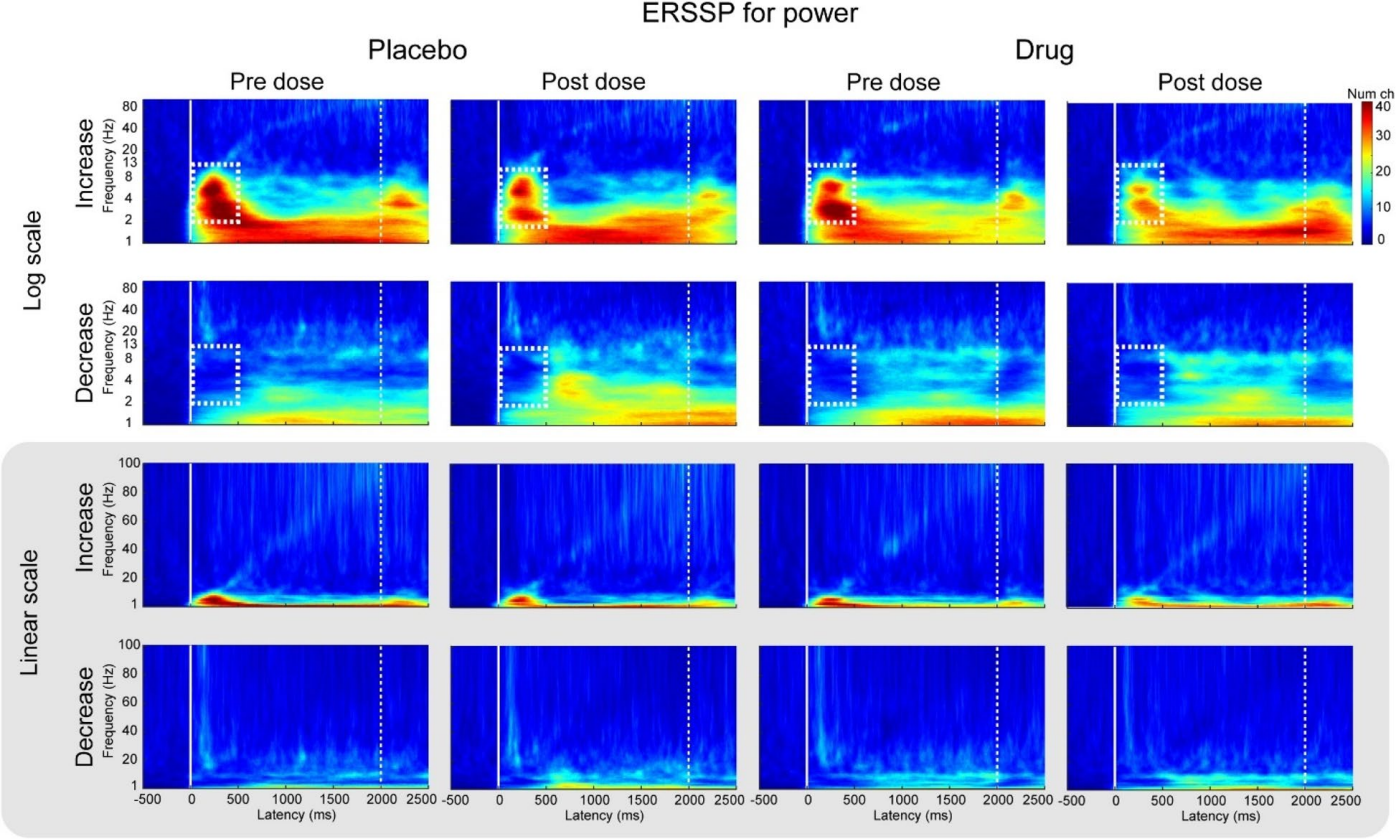
ERSSP analysis on power for Pre-Post comparison between the Placebo and Drug conditions using a 2 × 2 design. For the log-scaled plots, time–frequency ROI was set from 0 to 500 ms and from 2 to 13 Hz to test 2 × 2 interaction for increase (ERS) and decrease (ERD). None of the results reached statistical significance

We applied ERSSP analysis on the ERSP to compare the effect of Pre-Post conditions between the Placebo and Drug conditions on the VP, which employed a 2 × 2 factorial design. The target time–frequency ROI was from 0 to 500 ms and from 2 to 13 Hz. The results are shown in Fig. 15. Neither the interaction nor the main effect of Drug reached statistical significance.

##### ITC modulations on VP and 40-Hz peak are insensitive to the drug effect

**Fig. 16:**
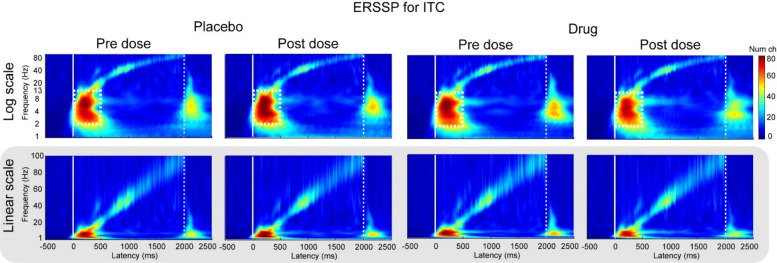
ERSSP analysis on ITC for Pre-Post comparison between the Placebo and Drug conditions using a 2 × 2 design. Two ROIs were set. For log-scaled plots, time–frequency ROI was set from 0 to 500 ms and from 2 to 13 Hz to test 2 × 2 interaction for increase (ERS) and decrease (ERD). For linear-scaled plots, time–frequency ROI was set at 40 Hz and 824 ms, which showed the maximum ITC at 40 Hz across time. None of the results reached statistical significance

We applied ERSSP analysis on the ITC to compare the effect of Pre-Post conditions between the Placebo and Drug conditions on the VP and 40-Hz peak, which employed a 2 × 2 factorial design. The target time–frequency ROI was from 0 to 500 ms and from 2 to 13 Hz for the VP and 824 ms at 40 Hz where the maximum ITC-ERSSP was observed. The results are shown in Fig. 16. Neither the interaction nor the main effect of Drug reached statistical significance in VPs or the 40-Hz ITC peak.

##### **Lab-Lab showed the highest Intraclass Correlation Coefficients (ICCs) in auditory ERPs**s

We tested ICC between Lab and Home conditions, Pre-dose only, for four EEG measures using ICC(A,1), which estimates absolute agreement for single measurements. The results are summarized in Table 4. The VP (N1-P1) peak amplitude difference yielded higher ICCs than other measures in most cases, followed by ERSSP-ITC_VP_. These two measures showed consistent values across comparisons. These two measures showed ICC values that ranged from 0.64 to 0.85, with Lab-Lab being generally higher than other pairs. We conclude that Lab-Lab reproducibility is better than Home-Home or Lab-Home pairs within our auditory-evoked data.

**Table 4:** Test–Retest Reliability (ICC) of Pre-dose Auditory EEG Measures

Measure	Lab-Lab	Home-Home	Lab-Home
VP (N1-P1) peak difference	0.85 [0.51 0.96], *p* = 0.0006	0.64 [0.05 0.90], *p* = 0.0185	0.72 [0.41 0.88], *p* = 0.0001
ERSSP-ERS_VP_ (ROI: 0–500 ms, 2–13 Hz)	0.65 [0.10 0.90], *p* = 0.0115	0.34 [−0.29 0.79], *p* = 0.1465	0.66 [0.32 0.85], *p* = 0.0006
ERSSP-ITC_VP_ (ROI: 0–500 ms, 2–13 Hz)	0.75 [0.08 0.94], *p* = 0.0151	0.66 [0.01 0.91], *p* = 0.0241	0.67 [0.33 0.86], *p* = 0.0005
ERSSP-ITC_40Hz_ (ROI: 824 ms, 40 Hz)	0.23 [−0.47 0.74], *p* = 0.2573	0.74 [0.13 0.94], *p* = 0.0106	0.37 [−0.10 0.70], *p* = 0.0591

*ICC confidence intervals in brackets*

### Relationships between performance changes and drug-induced power increases

**Fig. 17:**
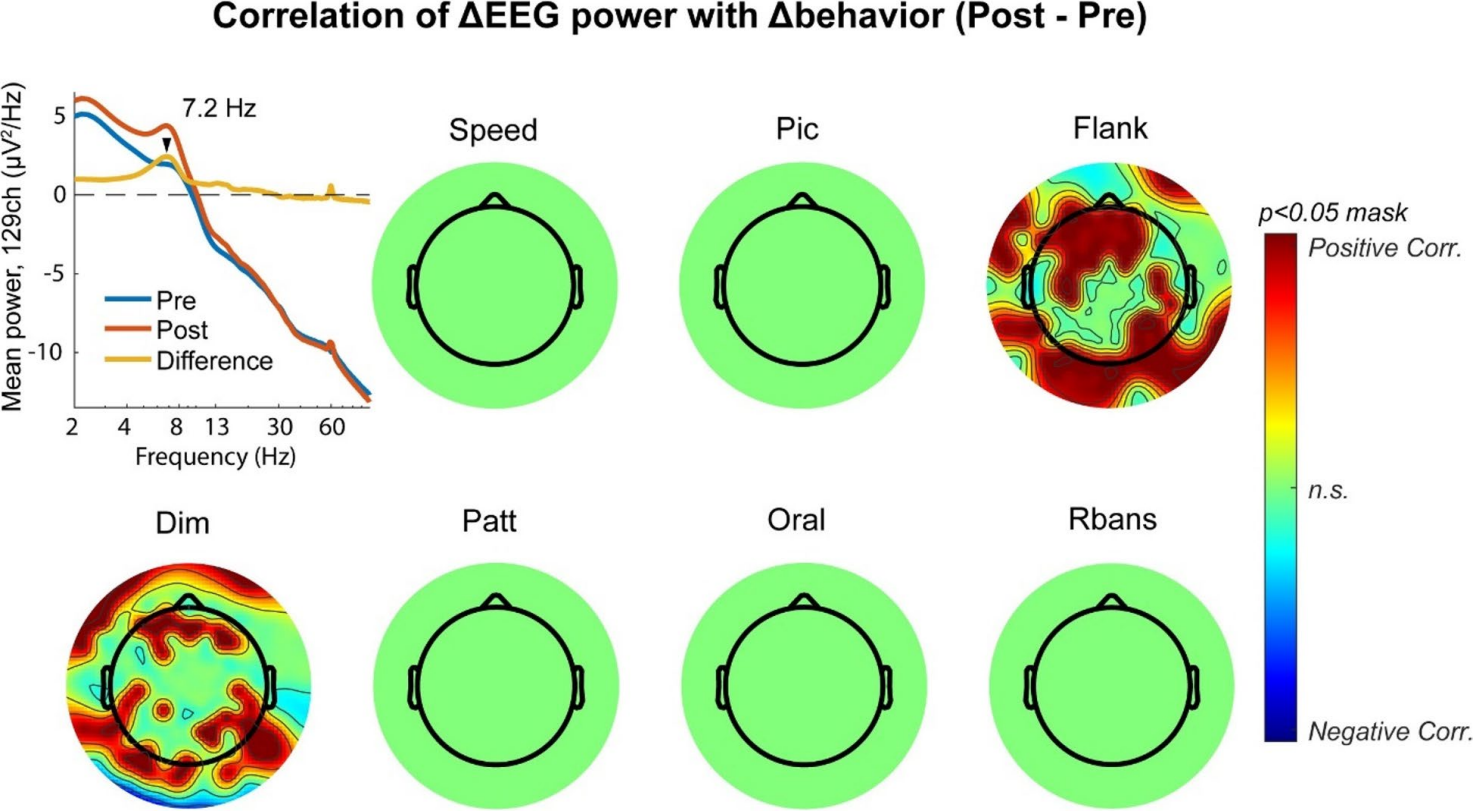
Correlation test between Post–Pre peak EEG power and Post–Pre cognitive test performance. Higher performances in the Flanker task and Dimension task were correlated with drug-induced peak power increase

We conducted a correlation analysis between change in cognitive performance and change in EEG power at the frequency of maximal drug effect, 7.2 Hz. The results are shown in Fig. 17. The analysis revealed that the improvement in performance in Flanker and Dimension tasks were correlated with increase in power at 7.2 Hz, while performance in other tasks was not correlated. The mean Pearson’s correlation coefficients across the significant electrode sites for Flanker and Dimension tasks were *r* = 0.34 and 0.21 (SD 0.36 and 0.32), respectively. Note that these results need to be interpreted with caution. Correlating a biomarker change with a non-significant behavioural change is known to be susceptible to spurious findings [23] and may be best used for hypothesis generation.

## Discussion

This study provides initial evidence that utilizing decentralized, home-based cognitive assessment and EEG is both feasible and reliable in adult males with FXS, while also evaluating the acute behavioral and neural effects of single-dose gaboxadol. Our results expand upon previous single-site biomarker-based clinical trials in FXS by demonstrating that critical measures, including cognitive batteries and relevant EEG measures such as resting-state spectral power and auditory-evoked potentials, can be collected consistently outside the controlled clinic setting, with between-location ICC values generally consistent with those found in the literature (see Tables 2–4). This finding is particularly important because challenges in traveling to a traditional research setting provide a barrier to broader participation in clinical trials among individuals with significant impairment, for whom upcoming treatments may provide the most benefit.

Pharmacodynamically, like in the *Fmr1* KO mouse [15] and similar to sleep studies in humans that found augmented theta power [27–29, 59], we found that gaboxadol was associated with increased theta and alpha power, with no significant effect on the gamma band. This result did not differ between home and lab, suggesting it is a robust indicator of gaboxadol’s effect on the brain and supporting the reliability of resting state EEG as a biomarker that can be collected both in-lab and in-home. Specifically, gaboxadol’s maximal effect was an augmentation of power in the 6–8 Hz range, with a concomitant shift in individual peak frequency in those for whom peaks could be detected, and a shift in the spectral exponent (SPEX) that suggests gaboxadol augmented inhibition in the brain.

Behaviorally, we observed a marginal increase in words remembered in the RBANS List Learning subtest following single-dose gaboxadol. While the RBANS findings suggest a numerical trend, the absence of statistical significance and potential baseline confounding suggest the results are best viewed as exploratory and hypothesis-generating**.** There were no effects of gaboxadol on the NIH Toolbox cognitive measures. Because of this, the reported correlation between the EEG power increase and performance on the Flanker and DCCS tasks should be presented with some caution. Correlating a biomarker change with a non-significant behavioral change in a small, exploratory study is potentially susceptible to spurious findings and should be considered, like with the RBANS, to be hypothesis-generating. Our lack of NIH Toolbox findings may be the result of increased noise in our data: while many studies screen datapoints on the examiner’s assessment of validity, our study did not.

The EEG results present a potential paradox. The data demonstrates that the drug engages its CNS target (evidenced by the robust increase in theta/alpha power) but fails to normalize the gamma band EEG abnormality that has been demonstrated repeatedly to characterize FXS neurophysiology. Additionally, acute single dose gaboxadol did not alter auditory-evoked EEG by any measure (ERP, ERSSP, ITC). This contrast suggests modulation of general tonic thalamo-cortical network activity, rather than correction of transient, stimulus-evoked processes known to be impaired in FXS.

Gaboxadol operates on extrasynaptic GABA_A_ δ-subunit–containing receptors and particularly the ${\alpha }_{4}{\beta }_{3}\delta$ subunit, which are predominantly expressed in the thalamus [45] and mediate tonic inhibition [52, 57]. Though the specific mechanism at work is undetermined, a recent preprint demonstrated that gaboxadol preferentially affected the mediodorsal thalamic nuclei (MD) during sleep [48], under review), which has dense connections with the posterior orbitofrontal cortex [18, 50]. The MD is involved in a broadly tuned olfactory pathway that is responsible for integrating input [62], distinct from the more finely tuned non-thalamic olfactory pathway [58]. The MD also has a connection to the amygdala, which provides emotional and reward-related value associated with sensory input [18, 62] and has previously shown hyperexcitability that was rescued by gaboxadol in a mouse model of FXS [41, 42].

We recently proposed a hyper-extralemniscal model of FXS [34], which highlights the role of supramodal, broadly-tuned extralemniscal thalamic pathway with diffuse cortical projections, in contrast to sharply-tuned, modality-specific lemniscal thalamic pathways. In line with this framework, our current findings may relate to the auditory extralemniscal thalamic pathway, which is primarily mediated by the medial division of the medial geniculate body (MGm). Like the MD’s olfactory pathway, the MGm’s auditory pathway is broadly tuned, terminates within the cortex, forms a dual pathway with a counterpart that is finely tuned, and has connections to the amygdala. From a dual system perspective [34, 55], the MD, if at work in the gaboxadol response, has more in common with the extralemniscal system, rather than the sensory-tuned lemniscal system. This would provide a potential anatomical basis for the observed increases in resting state power without changes to auditory-evoked activity.

We interpret these findings as supporting a model in which gaboxadol alters tonic activity in the thalamocortical loop, potentially via the MD, specifically engaging broadly tuned extralemniscal- (also known as non-specific-) like pathways. Although we do not have direct evidence to determine a specific mechanistic pathway, several observations converge: (1) modulation in the low-frequency band indicates the recruitment of thalamo-cortical loop; (2) the broad distribution of the maximum power increase across the whole scalp regions indicates recruitment of the extralemniscal thalamic system which is known to have spatially diffuse cortical and subcortical projections; (3) the absence of the modulation in auditory event-related potential indicates that the ‘saliency-encoding’ VPs remained intact [34, 36, 37, 54–56]. From these observations, we conclude that the spatiotemporal distribution of the effect of gaboxadol is broad and tonic. It modulates EEG states i.e., baseline, without affecting the transient ‘saliency network’ that is the source of the VPs [32, 36].

In this study, we quantified the empirical probability of observing an alpha peak in PSD across datasets and electrodes. We found that 30.2% (SD 13.2) of datasets show an alpha peak at a given electrode site. To validate this rate, we conducted an additional analysis on resting-state EEG data from the MPI Leipzig Mind-Brain-Body database [1]. We obtained a rate of 47.1% (SD 10.5). The details are reported in Supplement S2. The lower rate in our results may be explained by several factors: (1) participants watched silent movies during the eyes-open resting session in our data, while the participants in the LEMON dataset did not watch them,(2) our participants were a clinical population and half of the datasets were recorded at Home, while their participants were typically developing adults and were recorded in the Lab. Thus, the eyes-open resting session does not reliably produce a detectable alpha peak in a given electrode. This rate also depends on electrode locations. Our data confirmed that central and parietal regions show higher rates. Overall, we emphasize that Gaboxadol noticeably increased (161%) the number of electrodes with a detectable alpha peak, indicating the drug’s ability to increase oscillatory EEG signals.

This study should be interpreted in the context of several limitations. Our sample size was small, limiting statistical power and increasing susceptibility to both type I and type II error, particularly for exploratory analyses. While our inclusion of participants with diverse abilities increases the generalizability of our study, this contributes to increased variability in our behavioral outcomes and, in the case of verbal measures that some participants were unable to complete, further reduced the effective sample size for those measures. Additionally, no adjustments were made for multiple comparisons, and behavioral and EEG findings did not consistently converge, limiting our ability to characterize a consistent post-dose response profile. These limitations underscore the need for larger studies to clarify the relationship between GABA modulation and functional outcomes.

Taken together, the behavioral, EEG, and anatomical data support a model in which gaboxadol acts as a modulator of tonic thalamocortical activity in FXS. Importantly, these effects were observable both in-lab and in-home, suggesting that in-home data collection can provide an adequate replacement for clinic visits in participants who are unable to travel to a trial site. At the same time, these results do not provide strong evidence for a pro-cognitive or disease-modifying effect of single-dose gaboxadol in FXS. Furthermore, the interpretation of these acute, single-dose findings must be tempered by the potential effects of chronic administration. The acute effects may reflect transient arousal changes unlikely to persist with chronic dosing. While gaboxadol may have a unique profile that spares it from developing tolerance to its sleep-promoting effects, there is direct preclinical evidence that tolerance does develop for other functional effects, such as motor impairment.

## Supplementary Information

Supplementary Material 1.

## Data Availability

Due to the size and scope of the dataset, the data are not publicly hosted. The data are available from the corresponding authors upon reasonable request and in consultation with the study sponsor, HealX.
