# Supplementary material for: Gaboxadol increases resting theta and alpha power without affecting evoked responses in fragile X syndrome in a home-based setting
Source: J Neurodev Disord. 2026 May 8;18:36. doi: 10.1186/s11689-026-09700-5 (PMC13325590; doi:10.1186/s11689-026-09700-5)
Supplement: Supplementary file 1 — Supplementary Material 1. [file 11689_2026_9700_MOESM1_ESM.docx]

**Supplement**

**S1. Exclusion criteria**

Potential participants were deemed ineligible if they: (1) had any major medical comorbid condition deemed by a study physician as adding additional risk to the participant, such as refractory hypertension, kidney disease, or liver disease; (2) were diagnosed with diabetes or taking medication for diabetes; (3) had an unstable seizure disorder, including any seizure within six months prior to baseline and/or change in anti-convulsant treatment within the 60 days prior to baseline; (4) had changes in psychotropic or anti-convulsant medication (where taken for reasons other than seizure control) within 30 days prior to baseline; (5) had significant changes in any educational, behavioral, and/or dietary interventions in the month prior to baseline, other than normal changes due to the school calendar; (6) planned initiation of new, or modification of ongoing, interventions during the study; (7) were unable or unwilling to take oral medication in the form of a whole capsule; (8) consumed liver enzyme inducers or inhibitors including foods such as grapefruit, pomegranate, or star fruit within three days prior to any study visit; (9) had baseline laboratory assessments such as aminotransferase, aspartate aminotransferase, total bilirubin, or serum creatinine >1.5 x the upper limit of normal, or other clinically relevant laboratory abnormality; (10) had a clinically significant heart rate or blood pressure reading at baseline as judged by a study physician; (11) had received an investigational drug in any prior clinical study within 30 days or five half-lives (whichever is longer) prior to baseline.

**S2. Peak alpha presence rate**

We analyzed resting-state EEG data from the published Leipzig Study for Mind-Body-Emotion Interactions (LEMON) dataset (Babayan et al., 2019) to illustrate the general prevalence of detectable alpha peaks. Power spectra were averaged across electrodes and participants for each condition (eyes open, eyes closed), and peaks within the 4-13 Hz range were identified. Alpha peaks were detected in 76.4 ± 8.6% of participants during eyes-closed recordings and in 47.1 ± 10.5% during eyes-open recordings.


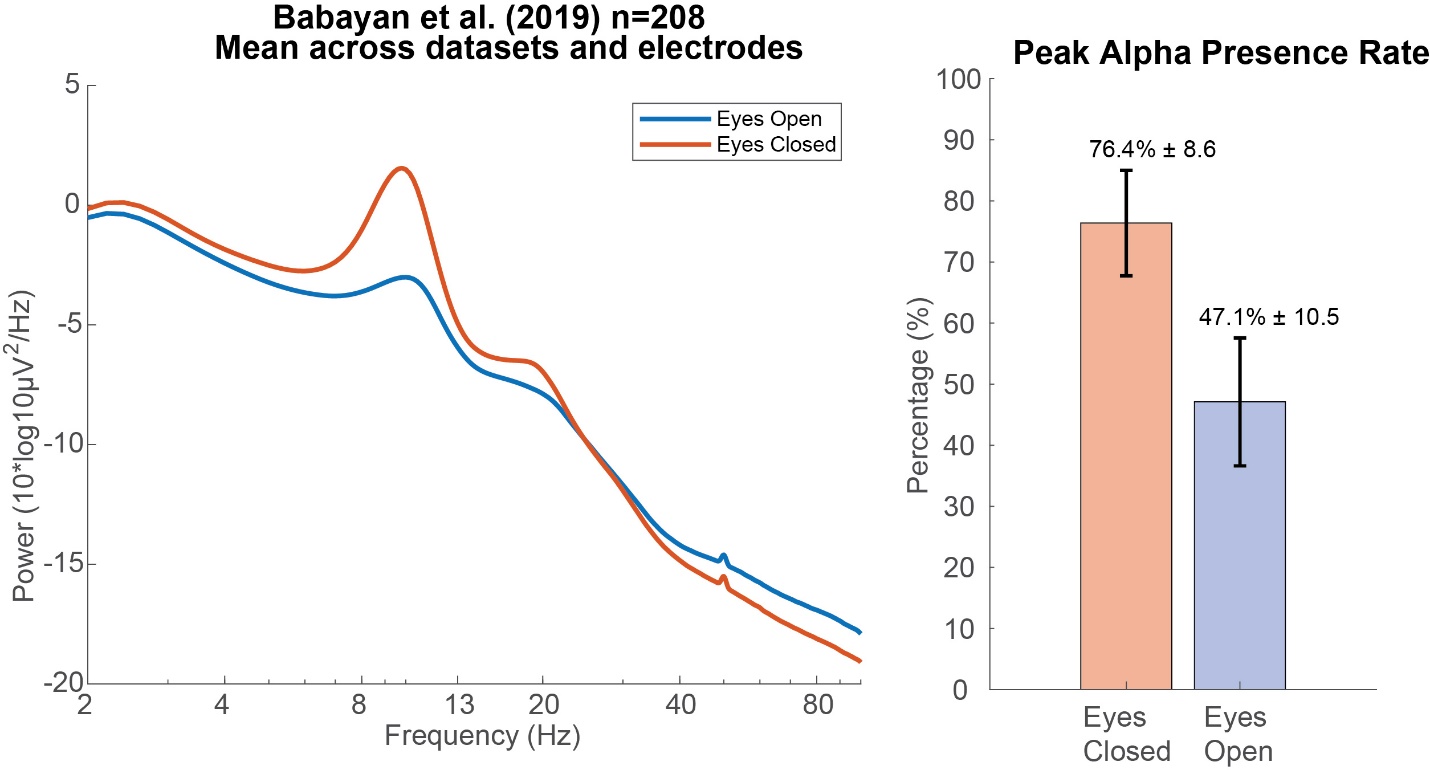


***Figure S1****. Alpha power and peak detection across eyes open and closed conditions. Left: Grand average power spectral density across all electrodes and participants (n = 208) from the published Leipzig Study for Mind-Body-Emotion Interactions (LEMON) dataset. A prominent alpha peak is observed, particularly in the eyes-closed condition. Right: Mean ± SD percentage of participants with a detectable alpha peak. Alpha peaks were more reliably detected in the eyes-closed condition (76.4 ± 8.6%) than in the eyes-open condition (47.1 ± 10.5%)*

**S3. Interaction between Drug-Placebo and Lab-Home**

In the main text, we focused on the main effect of Drug-Placebo. Here, we report significant interaction between Drug-Placebo and Lab-Home found in seven electrodes. These results were not interpretable.


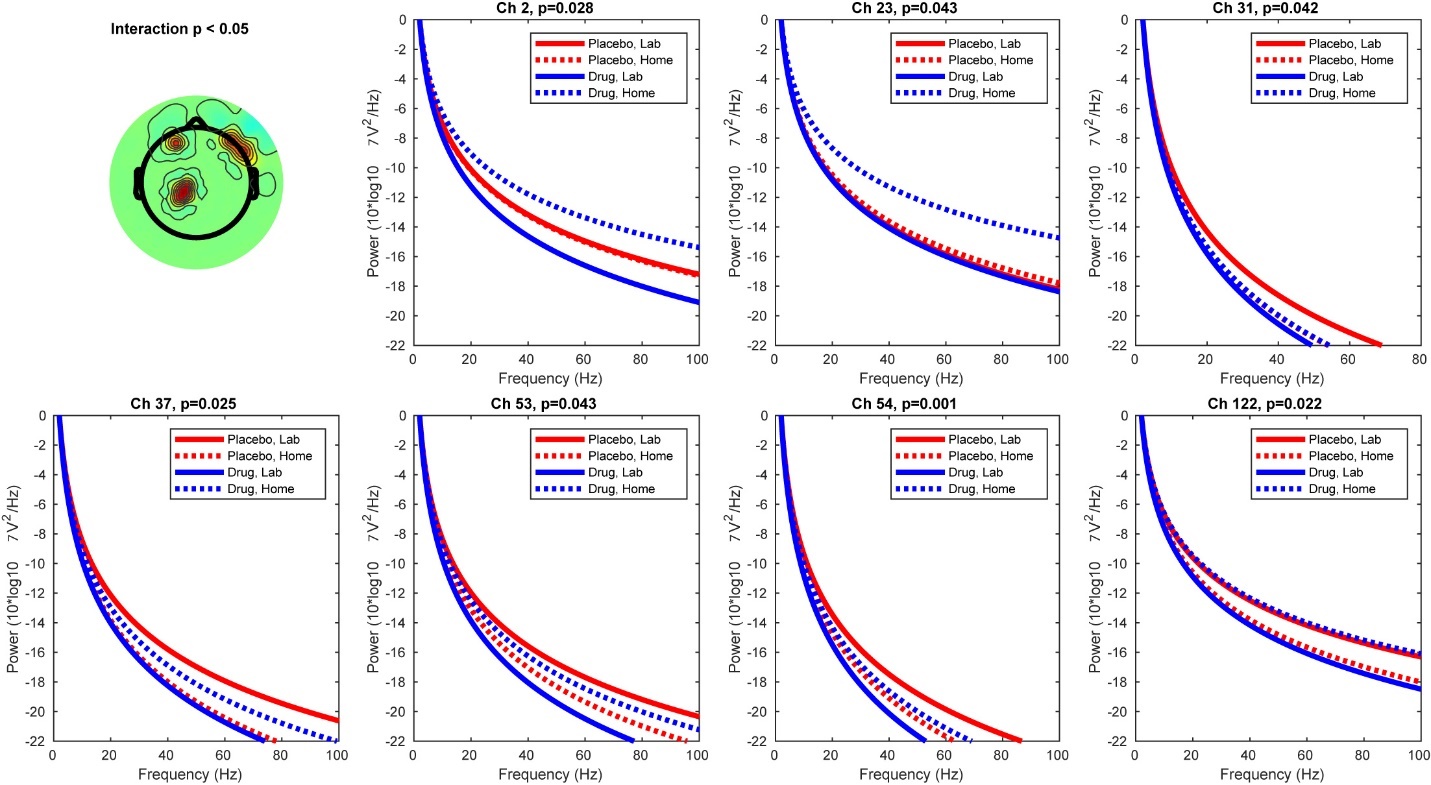


***Figure S2****.* Spectral exponent (SPEX) analysis for Lab-Home comparison for Placebo and Drug conditions using a 2 x 2 design. Seven electrode locations showed significant interactions.
